# rTg(Tau_P301L_)4510 mice exhibit increased VGlut1 in hippocampal presynaptic glutamatergic vesicles and increased extracellular glutamate release

**DOI:** 10.3389/fnsyn.2022.925546

**Published:** 2022-08-03

**Authors:** Erika Taipala, Jeremiah C. Pfitzer, Morgan Hellums, Miranda N. Reed, Michael W. Gramlich

**Affiliations:** ^1^Department of Physics, Auburn University, Auburn, AL, United States; ^2^Harrison School of Pharmacy, Auburn University, Auburn, AL, United States

**Keywords:** tau, fluorescence microscope methods, computational model, presynaptic transmission, cell culture, hippocampus, VGlut1, glutamate

## Abstract

The molecular pathways that contribute to the onset of symptoms in tauopathy models, including Alzheimer’s disease (AD), are difficult to distinguish because multiple changes can happen simultaneously at different stages of disease progression. Understanding early synaptic alterations and their supporting molecular pathways is essential to develop better pharmacological targets to treat AD. Here, we focus on an early onset rTg(Tau_P301*L*_)4510 tauopathy mouse model that exhibits hyperexcitability in hippocampal neurons of adult mice that is correlated with presynaptic changes and increased extracellular glutamate levels. However, it is not clear if increased extracellular glutamate is caused by presynaptic changes alone, or if presynaptic changes are a contributing factor among other factors. To determine whether pathogenic tau alters presynaptic function and glutamate release, we studied cultured hippocampal neurons at 14–18 days *in vitro* (DIV) from animals of both sexes to measure presynaptic changes in tau_P301L_ positive mice. We observed that presynaptic vesicles exhibit increased vesicular glutamate transporter 1 (VGlut1) using immunohistochemistry of fixed cells and an established pH-sensitive green fluorescent protein approach. We show that tau_P301L_ positive neurons exhibit a 40% increase in VGlut1 per vesicle compared to tau_P301L_ negative littermates. Further, we use the extracellular glutamate reporter iGluSnFR to show that increased VGlut1 per vesicle directly translates into a 40% increase in extracellular glutamate. Together, these results show that increased extracellular glutamate levels observed in tau_P301L_ mice are not caused by increased vesicle exocytosis probability but rather are directly related to increased VGlut1 transporters per synaptic vesicle.

## Introduction

Pathological hyperphosphorylation and aggregation of tau is a hallmark of neurodegenerative conditions known as tauopathies, including Alzheimer’s disease (AD), Parkinson’s disease (PD), Pick’s disease, and Frontotemporal dementia with Parkinsonism-17 (FTDP-17). While much of the work has focused on the role of pathological tau in disrupting postsynaptic signaling, there is growing interest in the role that pathological tau plays in presynaptic dysfunction (see Wu et al., 2021 for review). Using the rTg(TauP301L)4510 mouse model (hereafter called tau_P301L_ pos), we have previously demonstrated *in vivo* that P301L tau expression increases glutamate release in the hippocampus, which correlated with memory deficits, at a time when tau pathology was subtle and before readily detectable neuron loss (Hunsberger et al., 2014). This increase in glutamate release was concomitant with a 40% increase in hippocampal levels of the vesicular glutamate transporter VGlut1. Importantly, neither cognitive deficits nor the increase in glutamate release and VGlut1 levels were observed in the rTg(TauWT)21221 mouse model that expresses wild-type 4R0N human tau at concentrations equivalent to P301L human tau in tau_P301L_ pos mice, but without the P301L mutation and associated tau pathology (Hunsberger et al., 2014). This suggests that overexpression of tau *per se* did not mediate these alterations, which we have also previously demonstrated for postsynaptic alterations associated with P301L tau (Hoover et al., 2010). Moreover, reducing VGlut1 levels and glutamate release rescued cognitive deficits in tau_P301L_ pos mice (Hunsberger et al., 2015), suggesting the increased VGlut1 and/or glutamate release may represent potential therapeutic targets for the treatment of early-stage tauopathies.

Prior studies have also shown VGlut1 expression to be increased in the PS19 tauopathy mouse model in an age-dependent manner (Crescenzi et al., 2017), with levels increasing then decreasing with age. Likewise, while VGlut1 is decreased in AD patients, particularly in the later stages (Kashani et al., 2008), patients with early stages of mild cognitive impairment (MCI) demonstrate increased VGLUT (Bell et al., 2007). Together, these results suggest that the changes in VGlut1 expression may be age- or stage- dependent. This early increase in VGlut1 in MCI patients may help explain the hippocampal hyperexcitability that is predictive of the degree and rate of cognitive decline, as well as the conversion to AD, that has been previously observed in MCI patients (Mackenzie and Miller, 1994) and (see Toniolo et al., 2020 for review).

A major limitation to understanding the mechanisms of tau-mediated changes in presynaptic transmission has been the ability to separately distinguish mechanisms at the single vesicle level in a physiological approach. Prior *in vitro* cell cultures used immortalized cell lines or transfection of human tau (htau) in primary hippocampal cells (Zhou et al., 2017; McInnes et al., 2018; Siano et al., 2019), and have shown that tau mediates both an increase in VGlut1 expression and a decrease in vesicle exocytosis. *In vivo* approaches have also been able to measure increased VGlut1 expression and tau mediated reduction in synaptic transmission (Crescenzi et al., 2017), but could not directly measure the presynaptic exocytosis contribution from the post-synaptic response. Separating the consequences of increased VGlut1 from the decrease in synaptic transmission is essential because changes in VGlut1 expression have been shown to increase glutamate concentration per vesicle (Wilson et al., 2005) and changes in release probability (Herman et al., 2014). Thus, tau-mediated decreases in exocytosis and tau-mediated increases in VGlut1 expression levels can have counteracting effects on presynaptic transmission. These limitations have prevented a complete mechanistic understanding of the effect of tau-mediated alterations in VGlut1 levels on presynaptic release of glutamate *via* single vesicle exocytosis, which we address here.

Another limitation is separating the role of tau-mediated changes in frequency-dependent synaptic transmission. Normal synaptic transmission utilizes a complex train of stimulus from low frequencies (1 Hz) to high frequencies (100 Hz) (Abbott and Regehr, 2004; Klyachko and Stevens, 2006). Multiple studies of tauopathy models have shown hyperexcitable periods of higher frequency activity (>20 Hz) occurring for sustained bouts (Kazim et al., 2017). It is not clear if these periods of hyperexcitability are mediated by changes in a frequency-dependent alteration in presynaptic transmission. If tau mediates frequency-dependent release of glutamate, then this represents an important potential pathway of disease progression, because prior studies have demonstrated a glutamate-mediated exocytosis of tau as a potential mechanism for the trans-synaptic spread of tau pathology (Liu et al., 2012; Pooler et al., 2013; Yamada et al., 2014), and neuronal hyperexcitability represents one potential source for the pathogenesis of tauopathies (see Toniolo et al., 2020 for review). However, no careful study of frequency-dependent presynaptic glutamate release has been explored in cell culture models.

In the present study, we used nanometer fluorescence microscopy and computational analysis techniques to quantitatively explore how presynaptic vesicle VGlut1 is altered in a P301L tauopathy mouse model, as well as the resulting consequences on glutamate release, using primary hippocampal cell cultures of neurons grown on astrocytes. Presynaptic alterations were measured at 14–18 days *in vitro* (DIV) by isolating presynaptic transmission from postsynaptic response using established postsynaptic AMPA and NMDA receptor blockers during experiments (Voglmaier et al., 2006; Gramlich and Klyachko, 2017; Maschi and Klyachko, 2017; Maschi et al., 2018). We directly imaged single vesicle VGlut1 levels using the pH-sensitive GFP fluorescent protein pHlourin (Voglmaier et al., 2006; Maschi and Klyachko, 2017) and observed a 40% increase in VGlut1-pHluorin intensity per vesicle in tau_P301L_ pos neurons compared to tau_P301L_ neg neurons during stimulation. This increased VGlut1-pHluorin intensity was independent of stimulation frequency or single release probability per stimulation. Further, we imaged extracellular glutamate levels using the GFP fluorescent plasma membrane reporter iGluSnFR (Marvin et al., 2018) and observed that the increase VGlut1 per vesicle correlated with a 40% increase in iGluSnFR glutamate reporter intensity, supporting the hypothesis that increased VGlut1 transporters per vesicle results in extracellular glutamate released during stimulation. Finally, we used our results to computationally model the number of VGlut1 transporters per vesicle.

## Results

### Tau_P301L_ positive neurons exhibit increased VGlut1-pHluorin fluorescence intensity compared to tau_P301L_ negative neurons

**FIGURE 1:**
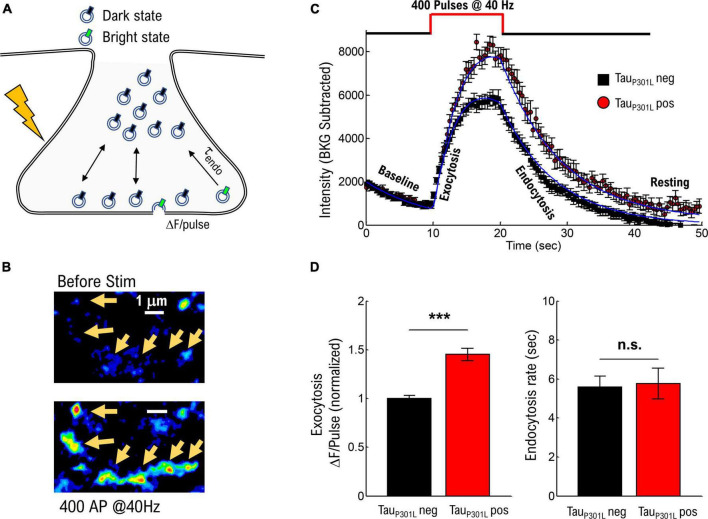
Tau_P301L_ pos neurons exhibit increased overall pHluorin-VGLUT1 intensity compared to tau_P301L_ neg neurons. **(A)** Representation of pHluorin-VGLUT1 intensity observed during presynaptic transmission. **(B)** Example of raw pHluorin-VGLUT1 data observed before and directly after a single bout of 40 Hz stimulation. **(C)** Comparison of pHluorin-VGLUT1 intensity before, during, and after 40 Hz stimulation for tau_P301L_ neg (Black) and tau_P301L_ pos (red) neurons. **(D)** Comparison of intensity/pulse and endocytosis rate for tau_P301L_ neg (Black) and tau_P301L_ pos (red) neurons. tau_P301L_ neg *N* = 257 presynapses, three samples from 2 litters; tau_P301L_ pos *N* = 160 presynapses, three samples from 2 litters. ***P < 0.001 for Mann–Whitney *U*-test. ns, not significant.

To determine if previously observed increased VGlut1 levels (Hunsberger et al., 2014) exists in tau_P301L_ pos hippocampal neurons compared to their transgene negative littermates (hereafter called tau_P301L_ neg), we used the established pH-sensitive fluorescent protein-based indicator VGlut1-pHluorin approach, which results in an increase in pHluorin intensity due to exposure of synaptic vesicles to the neutral pH of the extracellular environment (Figure 1A; Voglmaier et al., 2006; Maschi and Klyachko, 2017; Maschi et al., 2018). We fluorescently imaged transfected cells before, during, and after a single bout of electrical stimulation. We chose a single 10 s bout of electrical stimulation at a constant rate of 40 Hz to model a hyperexcitable state (Kazim et al., 2017) and drive a significant portion of the recycling pool to release. After stimulation, the resulting VGlut1-pHluorin intensity was then quantized for each vesicle released (ΔF/pulse, Figure 1A).

The observed VGlut1-pHluorin intensity exhibited an initial increase with stimulation (exocytosis, Figure 1C) followed by a saturation, and then a reduction in VGlut1-pHluorin intensity (endocytosis, Figure 1C), consistent with previous VGlut1-pHluorin studies in hippocampal neurons (Voglmaier et al., 2006; Maschi and Klyachko, 2017; Maschi et al., 2018). However, in this present study, tau_P301L_ pos exhibited an overall increase in VGlut1-pHluorin intensity as compared to tau_P301L_ neg controls (Figure 1C). Importantly, this change is not a consequence of a change in the density or number of presynapses that respond to stimulation as both tau_P301L_ neg and tau_P301L_ pos exhibit the same density, number, and size of presynapses during stimulation (Supplementary Figures 1A–D). Further, the difference in intensity is not a consequence of a different baseline intensity (Baseline Figure 1C) as both tau_P301L_ neg and tau_P301L_ pos exhibit statistically consistent baseline intensities (Supplementary Figure 1E).

Because each exocytosed vesicle immediately begins the endocytosis process, there are two potential contributions from vesicle recycling mechanics that could result in this observed difference in VGlut1-pHluorin intensity: either increasing intensity from exocytosis (ΔF/pulse, Figure 1A) or decreasing intensity from endocytosis (τ_endo_, Figure 1A; Chanaday and Kavalali, 2018). Using a continuum fit function (equation 1 in methods) to delineate the relative contributions, tau_P301L_ pos neurons showed an increase in ΔF/pulse 1.4x that of tau_P301L_ neg controls (*p* < 0.0001; Figure 1D, left), whereas the endocytosis rate was the same for both tau_P301L_ pos and tau_P301L_ neg neurons (*p* > 0.4; Figure 1D, right). The increase in intensity per stimulus (ΔF/pulse), but not endocytosis rate, in tau_P301L_ pos neurons suggests either a change in exocytosis mechanics or the amount of VGlut1 per vesicle or both.

To understand how tau_P301L_ pos neurons could exhibit increased VGlut1-pHluorin intensity compared to tau_P301L_ neg neurons, we developed a computational simulation model of the experimental results (Supplementary Figure 2; equation 2, methods). We then tested three possible pathways by which VGlut1-pHluorin intensity for tau_P301L_ pos neurons could be greater than that of tau_P301L_ neg neurons (Supplementary Figures 2C,D):

(i) tau_P301L_ pos presynapses exhibit a frequency-dependent increase (see section “Increased VGlut1-pHluorin intensity in tau_P301L_ pos neurons is independent of stimulation frequency and single release probability”).
(ii) tau_P301L_ pos release sites exhibit an overall 1.4x increase in release-probability (see section “Increased VGlut1-pHluorin intensity in tau_P301L_ pos neurons is independent of stimulation frequency and single release probability”).
(iii) tau_P301L_ pos vesicles exhibit 1.4x VGlut1-pHluorin intensity per vesicle compared to tau_P301L_ neg (see section “Tau_P301L_ pos neurons exhibit quantized increase in VGlut1-pHluorin intensity-per-vesicle as compared to tau_P301L_ neg neurons”).

### Increased VGlut1-pHluorin intensity in tau_P301L_ pos neurons is independent of stimulation frequency and single release probability

**FIGURE 2:**
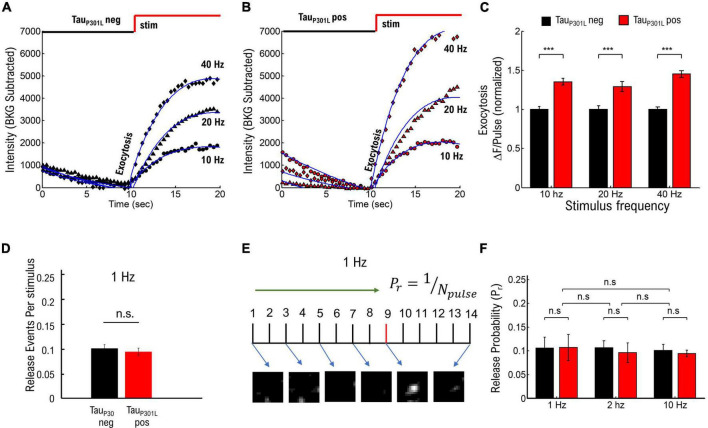
Frequency-dependent stimulation and single vesicle release probability do not contribute to increased tauP301L pos VGLUT1-pHluorin intensity. **(A)** Comparison of pHluorin-VGLUT1 intensity before (0–10 s) and during (10–20 s) stimulation as a function of stimulation frequency for tau_P301L_ neg neurons. **(B)** Comparison of pHluorin-VGLUT1 intensity before (0–10 s) and during (10–20 s) stimulation as a function of stimulation frequency for tau_P301L_ pos neurons. **(C)** ΔF/pulse parameter from eq. 1 fit to data (Blue Lines in **A,B**). Values at each stimulation frequency are normalized to the tau_P301L_ neg value for that frequency. **(D)** Number of released vesicles per stimulus pulse at 1 Hz for tau_P301L_ neg (black) and tau_P301L_ pos conditions (red). **(E)** Single Vesicle Release events as a function of Pulse Number for tau_P301L_ neg and tau_P301L_ pos conditions. **(F)** Release Probability as a function of stimulation frequency for tau_P301L_ neg (black) and tau_P301L_ pos conditions (red). Errors in **(C)** are twice the 95% confidence interval value of fits. ***P < 0.01; Statistical results from Mann–Whitney *U*. 10 Hz: tau_P301L_ neg *N* = 134 presynapses, one sample from 1 litters; tau_P301L_ pos *N* = 193 presynapses, two samples from 2 litters. 20 Hz: tau_P301L_ neg *N* = 615 presynapses, five samples from 3 litters; tau_P301L_ pos *N* = 151 presynapses, three samples from 2 litters. 40 Hz: tau_P301L_ neg N = 257 presynapses, three samples from 2 litters; tau_P301L_ pos *N* = 160 presynapses, three samples from 2 litters. 1 Hz: tau_P301L_ neg *N* = 43 vesicles, two samples from 2 litters; tau_P301L_ pos *N* = 37 vesicles, two samples from 2 litters. 2 Hz: tau_P301L_ neg *N* = 42 vesicles, three samples from 2 litters; tau_P301L_ pos *N* = 27 vesicles, three samples from 2 litters. 10 Hz: tau_P301L_ neg *N* = 112 vesicles, two samples from 2 litters; tau_P301L_ pos *N* = 111 vesicles, two samples from 2 litters. Errors in **(E)** are standard deviation of the mean, and statistical results from student *t*-Test. Errors in **(F)** are standard deviation of the mean, and statistical results from two-tailed KS-test of cumulative distributions. 1 Hz: tau_P301L_ neg *N* = 33 vesicles, two samples from 2 litters; tau_P301L_ pos *N* = 37 vesicles, three samples from 2 litters. ns, not significant.

Tau has been implicated in facilitating hyperexcitability in mouse models of AD as well as models of epilepsy (Palop et al., 2007; Roberson et al., 2007, 2011; Ittner et al., 2010; Peters et al., 2020), and it is possible that VGlut1-pHluorin intensity in tau_P301L_ pos mice may change as a function of neuronal stimulation. To establish if any stimulation frequency-dependent increase in vesicle release events exists in tau_P301L_ pos hippocampal neurons [e.g., pathway (i)], we performed VGlut1-pHluorin intensity experiments as a function of stimulus frequency using frequencies that range from moderate (10 Hz) to potential hyperexcitable frequencies (40 Hz). We again observed no difference in the endocytosis rate between any of the stimulation frequency results (*p*s > 0.35 for all tau_P301L_ neg/tau_P301L_ pos comparisons, Supplementary Figure 3). Surprisingly, we found that ΔF/pulse was consistently higher for tau_P301L_ pos neurons for all stimulation conditions (*p*s > 0.001; Figures 2A–C), suggesting that the increased VGlut1-pHluorin intensity observed in tau_P301L_ pos neurons is not frequency-dependent. Importantly, different stimulation frequencies were statistically different for the same condition (*p* < 0.001 for all frequency comparisons for tau_P301L_ neg, and *p* < 0.001 for all frequency comparisons for tau_P301L_ pos, Mann–Whitney-*U* tests). While these combined exocytosis and endocytosis results suggest that observed increases in tau_P301L_ pos VGlut1-pHluorin intensity is not a frequency-dependent release effect, the frequency-dependent results *do not* discount the possibility that tau_P301L_ pos neurons exhibit an overall increase in release probability independent of stimulation frequency.

We next focused on the hypothesis that single vesicle release probability is increased in tau_P301L_ pos neurons compared to tau_P301L_ neg controls (pathway (ii)) by using single vesicle release events at low stimulation frequencies, as previously published (Leitz and Kavalali, 2011; Maschi and Klyachko, 2017; Chanaday and Kavalali, 2018), to establish if overall tau_P301L_ pos release probabilities were higher than tau_P301L_ neg controls (Supplementary Figure 4). Briefly, we stimulated cultured neurons at a fixed frequency (1, 2, or 10 Hz) for 20–30 s, followed by a 20–40 Hz stimulation for 10 s to identify presynapses, and then quantified changes in VGlut1-pHluorin intensity during stimulation using a thresholding analysis method (see Supplementary Figure 4A). We then used two separate approaches to determine the probability of release for each stimulus pulse:

(i) We followed a previously established approach of measuring release probability with VGlut1-pHluorin (Chanaday and Kavalali, 2018) by counting the number of release events identified during 1 Hz stimulation and dividing by the number of pulses during stimulation (Figure 2D), which minimizes the possibility of false-positive counts due to a potential increase in intensity per vesicle hypothesized for tau_P301L_ pos. We then aggregated the fraction of release events per pulse for all presynapses to determine the release probability (Supplementary Figure 4B).
(ii) We counted the number of pulses until the *first release event* is observed for low stimulation frequencies, and we took the pulse just before the onset of intensity as the first release event (Figure 2E and Supplementary Figure 4D). We then aggregated the number of pulses before the first release event is observed for each condition to determine the release probability (Supplementary Figure 4E), and we defined the release probability as the inverse of the mean number of pulses until release (Pr = 1/number-of-pulses).

None of the resulting tau_P301L_ pos and tau_P301L_ neg vesicle release probabilities were statistically different from each other for the same frequencies or across frequencies (*p*s > 0.9, Figures 2E,F), suggesting there is no difference in vesicle release probability in tau_P301L_ pos neurons as compared to in tau_P301L_ neg neurons. Further, both approaches results in the same release probability measurement (*P*_r_ = 0.1), which is consistent with previously measured release probabilities using VGlut1-pHluorin (Leitz and Kavalali, 2011; Chanaday and Kavalali, 2018). Thus, the vesicle release probability results show that the increases in VGlut1-pHluorin intensity observed in Figure 1 are not caused by an overall increase in vesicle release probability in tau_P301L_ pos neurons as compared to tau_P301L_ neg controls. Together, these results suggest that neither proposed pathway (i) or (ii) are the mechanism by which tau_P301L_ pos VGlut1-pHluorin intensity is larger than tau_P301L_ neg.

### Tau_P301L_ pos neurons exhibit quantized increase in VGlut1-pHluorin intensity-per-vesicle as compared to tau_P301L_ neg neurons

If single vesicle VGlut1-pHluorin intensity alone reproduces observed differences between tau_P301L_ neg and tau_P301L_ pos neurons, as proposed in pathway (iii), then single vesicle release event intensities should be ∼1.4x the VGlut1-pHluorin intensity per tau_P301L_ neg vesicle. Before establishing intensity per vesicle, we sought to establish that only a single vesicle is released during a stimulus event since previous VGlut1-pHluorin experiments have established that presynapses can exhibit multiple vesicle release (Maschi and Klyachko, 2017; Maschi et al., 2018, 2021), which may also increase observed VGlut1-pHluorin intensities. We observed that the average width per release event, a measure of MVR, was the same for both tau_P301L_ pos and tau_P301L_ neg (Supplementary Figures 5A–C; *p* = 0.91), indicating that the increase in intensity in tau_P301L_ pos compared to tau_P301L_ neg is not due to an increase in multiple vesicle release.

**FIGURE 3:**
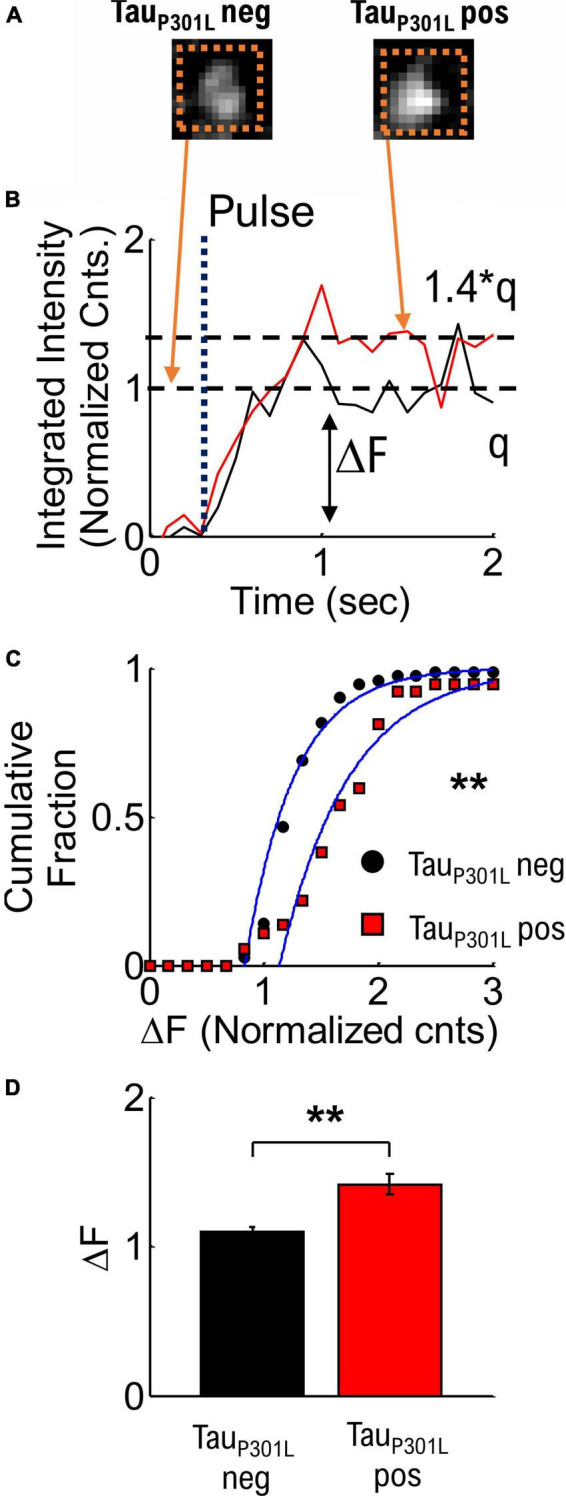
Quantized single vesicle pHlluorin-VGLUT1 intensity accounts for overall increased tau_P301L_ pos pHluorin intensity compared to tau_P301L_ neg neurons. **(A)** Example single vesicle events and integrated intensity analysis (Dashed Orange Boxes) for tau_P301L_ neg and tau_P301L_ pos neurons. **(B)** Sample integrated intensities traces for tau_P301L_ neg (Black line) and tau_P301L_ pos (Red Line) background subtracted and normalized to the average tau_P301L_ neg intensity. Single release events are observed after a stimulus pulse (Dotted Line labeled Pulse). Tau_P301L_ pos neurons exhibit larger intensity (ΔF) increase (1.4*q) than tau_P301L_ neg (q). **(C)** Cumulative fraction of aggregate single vesicle intensity [t] (ΔF) after stimulus (1 or 2 Hz) relative to background. All data normalized to average tauP301L neg intensity. Both curves fit with a cumulative fit function (Blue solid lines). **(D)** Mean ± SEM intensity per single vesicle release event for tauP301L neg and tauP301L pos from cumulative fractions in **(C)**. tauP301L neg *N* = 70 vesicles, five samples from 4 litters; tauP301L pos *N* = 56 vesicles, five samples from 4 litters. **P < 0.01, KS two-tailed test.

We next determined if the average VGlut1-pHluorin intensity per vesicle alone could explain observed increases in tau_P301L_ pos VGlut1-pHluorin intensity compared to tau_P301L_ neg. To determine single vesicle intensity, we used established integrated intensity of presynapses during low-stimulation frequencies (Leitz and Kavalali, 2011; Maschi and Klyachko, 2017; Chanaday and Kavalali, 2018), in combination with simple thresholding analysis to determine release events (Supplementary Figure 4A). Briefly, we used an integrated intensity box around single release events (Dotted Box, Figure 3A), and plotted the integrated intensity as a function of time (Figure 3B). We then quantified the difference between the background intensity and the average intensity after release (ΔF in Figure 3B). We note that our observed release events have the same rise-time (∼500 ms), dwell time (∼2 s, Supplementary Figure 4C), and decay time as previously observed in VGlut1-pHluorin measurements (Leitz and Kavalali, 2011; Chanaday and Kavalali, 2018).

To compare tau_P301L_ pos and tau_P301L_ neg single vesicle release event VGlut1-pHluroin intensity, we next aggregated the quantized integrated intensities (Figure 3C). The mean intensity for tau_P301L_ pos neurons was ∼1.4x times the tau_P301L_ neg intensity (*p* = 0.0046, Figure 3D). This result supports the hypothesis that the increase in VGlut1-pHluorin intensity in tau_P301L_ pos compared to tau_P301L_ neg is due exclusively to increased single vesicle VGlut1-pHluorin intensity. Further, since VGlut1-pHluorin intensity comes from a single fluorophore per VGlut1 transport, the increase in single vesicle VGlut1-pHluorin intensity is caused by an increase in the number of VGlut1 transporters per vesicle.

### Tau_P301L_ positive neurons exhibit increased VGlut1 expression compared to tau_P301L_ negative neurons

**FIGURE 4:**
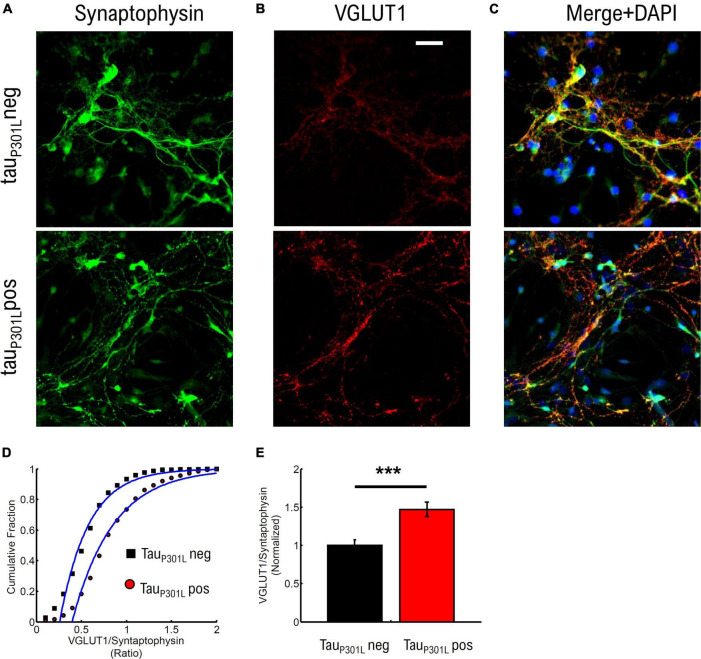
Tau_P301L_ pos neurons exhibit increased overall VGLUT1 expression compared to tau_P301L_ neg neurons. **(A)** Stained Synaptophysin intensity for tau_P301L_ neg (top), tau_P301L_ pos (bottom). **(B)** Stained VGLUT1 intensity for tau_P301L_ neg (top), tau_P301L_ pos (bottom). **(C)** Merged Synaptophysin (Green), VGLUT1 (Red), and DAPI (Blue) for tau_P301L_ neg (top), tau_P301L_ pos (bottom). **(D)** Cumulative distribution of VGLUT1/Synaptophysin intensity ratio for tau_P301L_ neg (black) and tau_P301L_ pos (red). **(E)** Mean ratio of VGLUT1/Synaptophysin intensity from fits in **(D)** and normalized to tau_P301L_ neg. Error-bars are errors of fit to data in **(D)**. ***P < 0.001, two-tailed student *t*-Test. tau_P301L_ neg *N* = 780, five samples from 3 litters; tau_P301L_ pos *N* = 1132, five samples from 3 litter.

To establish if tau_P301L_ pos neurons exhibit increased VGlut1 expression at 14–18 DIV cultures, we used immunostaining of synaptophysin and VGlut1 in cultured neurons (Figure 4) from tau_P301L_ pos and tau_P301L_ neg littermates. We then imaged puncta of synaptophysin intensity (Figure 4A), VGlut1 puncta intensity (Figure 4B), and accepted only co-localized puncta peaks to quantify relative puncta intensity (Figure 4C).

We compared the ratio of VGlut1/synaptophysin intensities to determine if an increase in VGlut1 is observed. The cumulative distribution of intensity ratios showed a clear increase in VGlut1 relative to synaptophysin for tau_P301L_ pos neurons compared to tau_P301L_ neg littermates (Figure 4D). To determine the relative increase in VGlut1, we then compared the fit mean values (Blue Lines Figure 4D) and observed a relative increase in VGlut1 of ∼1.48x (*p* < 0.001) in tau_P301L_ pos neurons (Figure 4E). We note that the synaptophysin intensity distribution varied less than 4% between samples of the same litter and across litters for both tau_P301L_ pos neurons compared to tau_P301L_ neg (Supplementary Figure 6).

These results show that VGlut1 expression is increased in tau_P301L_ pos mice compared to tau_P301L_ neg mice in cultured hippocampal neurons at 14–18 DIV. Further, the 40–50% increase in VGlut1 observed here is consistent with the 40% increase in VGlut1 levels previously observed in adult tau_P301L_ pos mice (Hunsberger et al., 2014, 2015).

### Tau_P301L_ pos neurons exhibit increased glutamate release as compared to tau_P301L_ neg neurons

**FIGURE 5:**
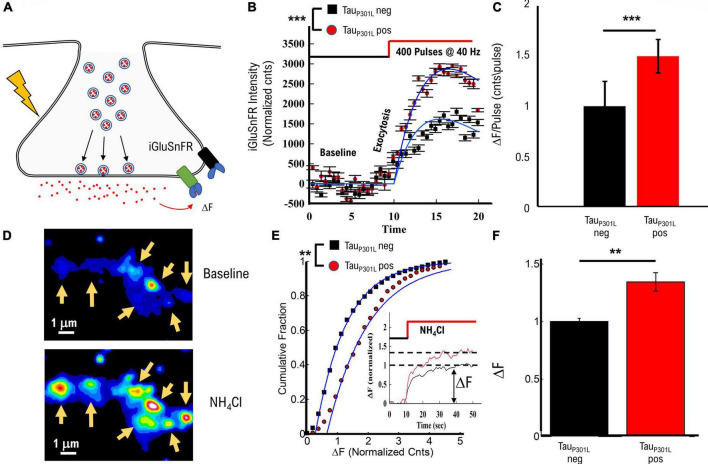
TauP301L pos exhibit increased overall iGluSnFR intensity compared to tauP301L neg controls. **(A)** Representation of iGluSnFR fluorescence observed during presynaptic transmission. **(B)** Example of raw iGluSnFR data observed before and directly after presynapse unloading induced by NH4Cl. **(C)** Comparison of iGluSnFR intensity before, during 40 Hz stimulation for tauP301L neg (Black) and tauP301L pos (red) neurons. **(D)** Comparison of intensity/pulse for tauP301L neg (Black) and tauP301L pos (red) neurons. **(E)** Quantitative comparison of NH4Cl induced iGluSnFR intensity. Inset shows example traces during exposure to NH4Cl. **(F)** Comparison of mean iGluSnFR intensity increase for tauP301L neg (Black) and tauP301L pos (red). 40 Hz: tauP301L neg *N* = 49 presynapses, from two samples; P301L *N* = 60 presynapses, from two samples; NH4Cl: tauP301L neg *N* = 375 presynapses, from two samples; P301L *N* = 526 presynapses, from two samples; ***P < 0.001, from Mann–Whitney *U*; **P < 0.01 for two-tailed *t*-Test. Error-bars are error of fits.

The concentration of glutamate per vesicle has been shown to be proportional to the number of VGlut1 transporters on the vesicle (Wilson et al., 2005). Increased glutamate concentration per vesicle would, in turn, result in an increased glutamate concentration in the synaptic cleft upon release. To establish the consequences of increased VGlut1 transporters per vesicle in tau_P301L_ pos neurons, we measured extracellular glutamate release using the established glutamate reporter iGluSnFR (Marvin et al., 2018) in which a membrane-bound GFP fluorescent protein fluoresces when glutamate exocytosed and binds the reporter (Figure 5A). Thus, the intensity of this reporter is proportional to the amount of glutamate released during synaptic transmission (Marvin et al., 2018).

To determine the effects of increased VGlut1 on stimulated release of extracellular glutamate per synapse, we used the same single bout of 40 Hz stimulation approach for Vglut1-pHluorin as used in Figure 1. Intensity increased during 40 Hz stimulation for both tau_P301L_ neg and tau_P301L_ pos (Figure 5B), but tau_P301L_ pos exhibited a significantly greater overall intensity consistent with observed changes in VGlut1-pHluorin (Figure 1C). We then used the same continuum model fit used for VGlut1-pHluorin (eq. 1) to quantify the difference observed in the iGluSnFR intensity. We found that tau_P301L_ pos neurons exhibited a similar increase in iGlSnFR intensity compared to tau_P301L_ neg (*p* < 0.001, Figure 5C).

To determine the effects of increased VGlut1 on overall extracellular glutamate release per synapse and support our 40 Hz stimulated results, we applied NH_4_Cl in the bath solution during imaging, which has been shown to alkalinize internal presynaptic compartments, release internal Ca^2+^, partially de-polarize the membrane and drive vesicles to exocytose at the membrane (Figure 5D; Ganguly et al., 2015; Lazarenko et al., 2017; Chanaday and Kavalali, 2018) resulting in a significant increase in iGluSnFR intensity (Figure 5D). Importantly, the overall spatial scale of intensity increase is the same as observed in VGlut1-pHluorin intensity (Figure 1B), which supports that our glutamate measurement is localized to the presynaptic regions, consistent with previous iGluSnFR studies in hippocampal cell cultures (Marvin et al., 2018). We quantitatively compared intensity increase for iGluSnFR over the same spatial range surrounding identified presynapse locations as used in pHluorin to proportionally compare any changes.

We observed a significant increase in iGluSnFR intensity in tau_P301L_ pos as compared to tau_P301L_ neg controls (Figure 5E). Mean tau_P301L_ pos intensity increased by ∼1.4x compared to tau_P301L_ neg controls (*p* = 0.0025; Figure 5F). This increased iGluSnFR intensity is proportional to both the overall increase in pHluorin intensity during stimulation (Figure 1 and Supplementary Figures 1C,D) and increased single vesicle intensity (Figure 3). Thus, the iGluSnFR results support the hypothesis [pathway (iii)] that increased VGlut1 transporters per vesicle result in an increased extracellular glutamate release into the synaptic cleft.

### Estimated increase in number of VGlut1 transporters per vesicle

The results from this study provide key insights into presynaptic changes that may support observed cognitive changes during disease progression. The results here show that the number of vesicles released during synaptic transmission do not change in tau_P301L_ pos neurons, but the number of VGlut1 transporters is increased. We can now use these results to estimate how many VGlut1 transporters per vesicle exist in tau_P301L_ pos and how many vesicles are released during synaptic transmission, which can then be used to better model changes that occur later in disease progression.

**FIGURE 6:**
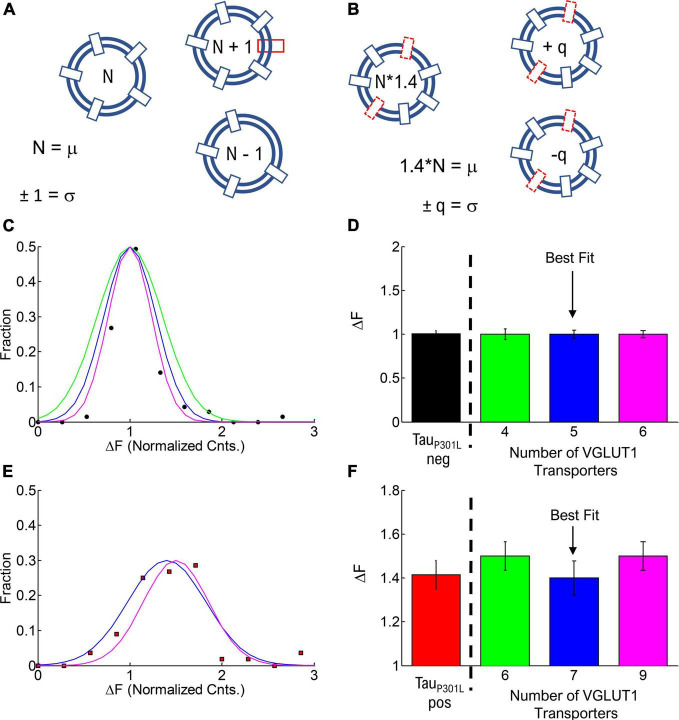
Estimate of number of VGLUT1 transporters per vesicle. **(A)** Cartoon model of the number of VGLUT1 transporters per vesicle (N) and the variance in number (σ). **(B)** Cartoon model of the increase in number of VGLUT1 transporters in tauP301L pos (N*1.4) and the variance in number (σ). **(C)** Fraction of single vesicle intensities per vesicle in tauP301L neg (Solid circles) and modeled integer number of VGLUT1 transporters per vesicle of 4 (Green), 5 (Blue), and 6 (Magenta), with each having a variance of 1 per vesicle. **(D)** Normalized intensity per vesicle and variance for all modeled options of tauP301L neg (Colors same as **C**). **(E)** Fraction of single vesicle intensities per vesicle in P301L (Solid squares) and modeled integer number of VGLUT1 transporters per vesicle equal to the nearest integer increase above tauP301L neg (6 = 1.5*4, Green), (7 = 1.4*5, Blue), and (9 = 1.5*6, Magenta), with each having a variance of 1 per vesicle. **(F)** Normalized intensity per vesicle and variance for all modeled options of tauP301L pos (Colors same as **E**).

Since single vesicle VGlut1-pHluorin intensity is caused by the intensity per VGlut1 transporter, it is not physically possible for tau_P301L_ pos single vesicles to have 1.4x the number of VGlut1 transporters as they must be whole integers. We can then use the single vesicle intensity distributions to estimate the number of VGlut1 transporters per vesicle in both tau_P301L_ neg and tau_P301L_ pos. We first consider that the integer number of VGlut1 transporters per vesicle in tau_P301L_ neg (N, Figure 6A) results in the mean single vesicle intensity observed, and the distribution of number of VGlut1 transporters equals the variance in observed intensity (σ, Figure 6A). The observed increase in the tau_P301L_ pos Gaussian distribution of intensity must then be constrained to an integer number (1.4*N, Figure 6B), with an integer variance in the number per vesicle (σ, Figure 6B). These constraints leave a limited number of potential options for the number of VGlut1 channels that satisfy both the tau_P301L_ neg and tau_P301L_ pos distributions (Table 1).

**TABLE 1:** Estimated change in number of VGlut1 transporters per vesicle in tau_P301L_ pos and tau_P301L_ neg conditions. Best estimate in the number of transporters was determined by residuals square fit to experimental results (Figures 6C–F).

	Condition	*N*	+/−	Normalized	Variance	Residuals
Too low	tau_P301L_ neg	4	1	1	0.25	0.088
	tau_P301L_ pos	6	2	1.5	0.5	
Best	tau_P301L_ neg	5	1	1	0.2	0.078
	tau_P301L_ pos	7	3	1.4	0.6	
Too high	tau_P301L_ neg	6	1	1	0.166667	0.134
	tau_P301L_ pos	9	3	1.5	0.5	

We first modeled the tau_P301L_ neg single vesicle condition to find the minimum number of VGlut1 transporters that would satisfy the observed result (Figures 6C,D). We found that a minimum of 4 transporters with a variance of 1 and a maximum of 6 transporters with a variance of 1 satisfied the constraints observed experimentally; below or above those values lead to values outside previously physically observed limits of VGlut1 transporters (between 5 and 12 transporters per vesicle) (Takamori et al., 2006).

We then multiplied the number of VGlut1 transporters per tau_P301L_ neg vesicle (4, 5, 6) by the factor that resulted in the next nearest integer number of vesicle per transporter; for example, an *N* = 4 transporters for tau_P301L_ neg would be multiplied by 1.5* to get an integer increase to 6 transporters per tau_P301L_ pos vesicle. We then found that for tau_P301L_ neg VGlut1 to have both *N* = 4, 6 would require a factor of 1.5 times to get the nearest integer of VGlut1 of 6 (Green, Figures 6E,F) and 9 (Magenta, Figures 6E,F) respectively in tau_P301L_ pos. The best fit result was the model of *N* = 5 VGlut1 transporters for tau_P301L_ neg and *N* = 7 = 1.4*5 VGlut1 transporters for tau_P301L_ pos (Blue, Figures 6E,F).

## Discussion

In this present study, we found tau_P301L_ pos mice exhibit increased VGlut1-pHluorin intensity compared to tau_P301L_ neg littermates using the pH-sensitive fluorescent protein VGlut1-pHluorin (Figure 1). We hypothesized that there were several pathways that could result in increased tau_P301L_ pos pHluorin intensity (Supplementary Figure 2). We found that the increased pHluorin intensity was not stimulation frequency dependent (Figure 2), and that the release probability was the same for both tau_P301L_ pos and tau_P301L_ neg (Figure 2). After establishing that only single vesicles released for each stimulation pulse (Supplementary Figure 5), we observed that each single release event exhibited an ∼1.4x VGlut1-pHluorin intensity for tau_P301L_ pos as compared to tau_P301L_ neg (Figure 3) and that tau_P301L_ pos mice exhibited increased VGlut1 levels compared to tau_P301L_ neg littermates at 14–18 DIV (Figure 4). Finally, we measured extracellular glutamate released using the fluorescent reporter iGluSnFR, and found that tau_P301L_ pos neurons exhibit a 1.4x increase in glutamate released as compared to tau_P301L_ neg littermates (Figure 5). We conclude that increased single vesicle pHluorin intensity is caused by an increase in VGlut1 per vesicle, and the increased VGlut1 results in increased extracellular glutamate released during synaptic transmission.

One major problem in understanding how tau mediates presynaptic pathological changes during disease progression is inconsistent results from different methodological approaches. Further, altered VGlut1 levels and tau mislocalization in the presynapse can have confounding effects on presynaptic transmission. Therefore, it is essential to put the results from this study in the broader context of previous studies on tauopathy effects on presynaptic transmission.

First, our cellular culture approach differs from other cell culture approaches in keyways. We studied tau_P301L_ pos neurons grown on tau_P301L_ pos astrocytes and compared their differences to tau_P301L_ neg neurons grown on tau_P301L_ neg astrocytes. Previous studies have used immortalized hippocampal cells or neuroblastoma (SHSY5Y) cell lines to study tau-induced VGlut1 levels, which have not been shown to be directly related to the time-course of tauopathy in P301L (Siano et al., 2019). Alternatively, primary hippocampal neurons in htau mouse lines have been grown directly on treated glass coverslips (Zhou et al., 2017; McInnes et al., 2018), but their results are physiologically limited because previous studies have also shown that neuron-astrocyte interactions can be a contributing factor in cell cultures of tauopathy models (Sidoryk-Wegrzynowicz et al., 2017). Further, cultured hippocampal neuron studies used transfection with a htau mutation to show a significant reduction in presynaptic transmission (Zhou et al., 2017; McInnes et al., 2018; Siano et al., 2019); however, it has not been shown how acute introduction of tau is directly related to the normal time-course of neurodegeneration. Therefore, our approach provides the advantage of measuring tau-mediated presynaptic vesicle release changes during their development, and in the presence of their appropriate astrocytic growth factors.

Second, our observation of VGlut1 and our isolation of presynaptic transmission from postsynaptic signaling allows for a direct measurement of the contribution of VGlut1 in presynaptic transmission in tauopathy models. Previous age-dependent *in vivo* mouse model studies have been able to correlate changes in VGlut1 expression levels and their effect on calcium (Wu et al., 2021) or glutamate (Hunsberger et al., 2014, 2015), but these studies could not directly measure if VGlut1 levels mediated increased extracellular glutamate; this is particularly important because changes in VGlut1 levels have been shown to directly affect presynaptic release probability (Wilson et al., 2005, p. 1). Direct measurements of VGlut1 levels on synaptic transmission using *in vitro* cell culture methods were inconsistent with *in vivo* results (Siano et al., 2019), possibly due to the limitations of the cell culture approach used, as outlined above. The approach in our study directly measures VGlut1, extracellular glutamate, and blocks the post-synaptic response, thereby allowing for an isolated measurement on presynaptic transmission. However, we note that an increase in the variance of the number of VGlut1 transporters per vesicle (Figure 6), could have a contributing factor to differences in post-synaptic response at the single quantal release level. This possibility could be pursued in future combined studies of pre/post-synaptic transmission in our model.

Finally, our measurement of single vesicle VGlut1 levels and single vesicle presynaptic release probability separates the confounding roles of VGlut1 and vesicle release. It is not clear if presynaptic VGlut1 levels increase prior to tau-mediated decreases in presynaptic transmission or if the two occur simultaneously. Previous *in vitro* studies of tau binding to single vesicles resulting in a reduction in presynaptic transmission did not measure VGlut1 levels and were done using htau transfections (Zhou et al., 2017; McInnes et al., 2018). Alternatively, *in vitro* or *in vivo* cell culture VGlut1 expression level experiments showed a change in extracellular glutamate levels but did not directly measure single vesicle presynaptic transmission (Hunsberger et al., 2015).

## Conclusion

Confounding tau-mediated changes in presynaptic mechanisms make it difficult to distinguish presynaptic contributions to disease progression in AD. Previous studies have shown that tau mediates increases in VGlut1-transporters per vesicle and reductions in presynaptic release probability, however, no study has determined if these changes occur simultaneously or separately. Our results show that increases in VGlut1-transporters per vesicle can occur prior to tau-mediated reduction in release probability. Further, our results show that increased VGlut1-transporters per vesicle result in an increase in extracellular glutamate release, supporting the hypothesis that increased presynaptic glutamate release mediates hyperexcitable states in AD.

## Materials and methods

### Continuum model of Vglut1-pHluorin/iGluSnFR intensity with electrical stimulation

There are two potential contributions from vesicle recycling mechanics that could result in the differences between tau_P301L_ pos and tau_P301L_ neg intensity. Because each exocytosed vesicle immediately begins the endocytosis process, observed VGlut1-pHluorin intensity simultaneously includes increasing intensity from exocytosis (ΔF/pulse, Figure 1A) and decreasing intensity from endocytosis (τ_endo_, Figure 1A; Chanaday and Kavalali, 2018). To delineate their relative contributions, we developed a continuum fit function that can distinguish exocytosis and endocytosis parameters, while reproducing observed intensities:


(1) $$\begin{array}{l} \Delta F\left(t\right)=F_{BKG^{e}}{}^{-\frac{t}{\tau BKG}}\\ +\{\left(\Delta F_{pulse}*f*t\right)\left[\Theta \left(t-10\right)-\Theta \left(t-20\right)\right]\left(\Delta F_{pulse}*f*20\right)\\ {}[\Theta \left(t-20\right)\}*e^{-\frac{t-10}{\tau_{endo}}} \end{array}$$

where F_BKG_ is the starting background intensity, τ_BKG_ is the background photobleaching rate constant, ΔF_Pulse_ is the pHluorin intensity increase per pulse, f is the stimulation frequency, Θ is the Heaviside function, τ_endo_ is the endocytosis rate constant.

This continuum function reproduced the observed VGlut1-pHluorin intensity with time for both tau_P301L_ pos and tau_P301L_ neg neurons (Blue Solid Lines, Figure 1C). From this analysis, we were able to separately quantify the intensity increase per stimulus (ΔF_Pulse_, Left Panel, Figure 1D) and the endocytosis rate (τ_endo_, Right Panel, Figure 1D).

### Computational model of Vglut1-pHluorin intensity with electrical stimulation

Our model is based on the established binomial model of presynaptic transmission (Reid and Clements, 1999; Scheuss and Neher, 2001; Lanore and Silver, 2016). The established model predicts a presynaptic response to a single stimulus as:


(2) R=pq

where R is the response, p is the probability of release per stimulus, and q is the post-synaptic response.

Here we modify the traditional model to reproduce Vglut1-pHluorin intensity as (See Supplementary materials):


(3) $$\text{I}\,\left(△\,\text{t}\right)=△\,{\text{Fe}}^{-t/\tau }*\text{p}+{\text{I}}_{\text{BKG}}\,\left(△\,\text{t}\right)\,I\,\left(△\,\text{t}-1\right)$$

where each simulation time-step (Δt) includes the intensity from the previous simulation time-step, a total background intensity (I_BKG_, equivalent to eq. 1), and a probabilistic increase (p) in intensity given by a per vesicle count (ΔF, equivalent to eq. 1) from each stimulus pulse, and each vesicle has the same endocytosis rate (τ, equivalent to eq. 1).

In the present study, we used parameters for endocytosis and release probability based on experimentally determined values obtained in this study. We required that all vesicles have the same endocytosis rate (τ = 7 sec^–1^), found from fits in eq. 1. To model tau_P301L_ neg single vesicle release probability per stimulus pulse, we used the experimentally observed release probability (*P* = 0.1) found in the present study (Figure 4D). We allowed the intensity per vesicle and overall release probability per site to change in order to model observed tau_P301L_ pos intensity.

To confirm the validity of this model, we first compared its results to observed tau_P301L_ neg VGlut1-pHluorin intensity. This model reproduced observed experimental tau_P301L_ neg VGlut1-pHluorin intensity (Solid line through tau_P301L_ neg, Supplementary Figure 2) with equivalently good agreement as the continuum model in eq. 1 (Figure 1C). Further, this model results in the same quantitative intensity per pulse as the experimentally observed (Left Panel Figure 2D). Thus, this model provides a sufficient representation of observed presynaptic release for modeling tau_P301L_ pos VGlut1-pHluorin.

The first pathway is tested experimentally in the following section “Tau_P301L_ pos neurons exhibit quantized increase in VGlut1-pHluorin intensity-per-vesicle as compared to tau_P301L_ neg neurons.” The second pathway, modeled computationally as an increased *p*-value (p, eq. 3), reproduces observed tau_P301L_ pos intensity (Solid Line, Supplementary Figure 2); this pathway is experimentally tested in section “Tau_P301L_ positive neurons exhibit increased VGlut1 expression compared to tau_P301L_ negative neurons.” The third pathway, modeled as an increase in the VGlut1-pHluorin intensity per vesicle (ΔF, eq. 3), results in the same observed tau_P301L_ pos VGlut1-pHluorin intensity (Dashed Line overlaps solid line, Supplementary Figure 2).

### Single vesicle release measurements

To measure single vesicle release events we used a standard threshold analysis (Supplementary Figure 4) to measure vesicles at low stimulation. Our approach is similar to a previously established method of observing single Vglut1-pHluorin intensity events at low stimulation frequencies (Maschi and Klyachko, 2017). Briefly, we stimulate cultured neurons at a fixed frequency (1, 2, or 10 Hz) for 30 s and observe VGlut1-pHluorin intensity during stimulation, followed by a higher frequency stimulation to confirm presynaptic location. We count the number of pulses until the first release event is observed (Figures 4A,B). For low stimulation frequencies (1 or 2 Hz), a single release event was typically observed for several frames before endocytosis occurred (Figure 4A); whereas, for a moderate stimulation frequency (10 Hz), recurring events resulted in increasing intensity after the initial release (Figure 4B). Consequently, we take the pulse just before the onset of intensity as the first release event (Green Arrow, Figure 4A). We then aggregate the number of pulses before the first release event is observed for each condition to determine the release probability (Red arrows, Figures 4A–C). Lastly, we then define the release probability as the inverse of the mean number of pulses until release (Figure 4A):


(4) Pr=1/number⁢-⁢of⁢-⁢pulses

We note that the definition in our present study differs from previous VGlut1-pHluorin measurements, where a single pulse is initiated and a small number of release events are counted (Maschi and Klyachko, 2017; Maschi et al., 2018). However, this approach presents an effective quantitative comparison between tau_P301L_ pos and tau_P301L_ neg vesicle release probability because they are being compared using the same metric.

### Single vesicle intensity analysis

To determine integrated intensity changes in single vesicle release events, we quantified the average intensity for the first five frames above our threshold (Supplementary Figure 4) and subtracted the average intensity for five frames just before the threshold. Aggregate cumulative distributions for all measured release event intensities (ΔF) was then compared for tau_P301L_ pos (Red Squares, Figure 3C) and tau_P301L_ neg (Black Circles, Figure 3C). We then fit both distributions to a cumulative fit function (Blue Lines, Figure 3C), and compared the resulting fit means to quantitatively determine the difference between tau_P301L_ pos and tau_P301L_ neg.

### Imaging media for VGlut1-pHluorin and iGluSnFR experiments

All experiments were performed with samples between 34 and 37°C within a whole-microscope incubator (OKO Labs) at DIV14–19 (As described in Supplementary materials). During experiments, cultures were perfused with bath solution (140 mM NaCl, 2.5 mM KCl, 2 mM CaCl_2_, 4 mM MgCl_2_, 10 mM HEPES, 2 mM Glucose, 50 mM DL-AP5, 10 mM CNQX, pH adjusted to pH 7.4), consistent with previous VGlut1-pHluorin experiments (Leitz and Kavalali, 2011; Chanaday and Kavalali, 2018; Maschi et al., 2018). Solutions were heated using a temperature controller attached to a multi-line solution heater (Warner Instruments). Ammonium Chloride solutions contained the above solution mixture plus 50 mM NH_4_Cl and pH-balanced to 7.4.

### Immunocytochemistry data analysis

Immunohistochemistry samples were prepared and imaged as described in Supplementary materials. Images were analyzed by first subtracting using a 30-pixel rolling ball radius in ImageJ; followed by a 2-pixel Gaussian-blur to reduce noise. Peak intensity (*x,y*) locations were then identified separately for each channel (Synaptophysin, Figure 4A; and VGlut1, Figure 4B). Peak intensity positions that co-localized in both channels to within 1 pixel (<367 nm) were then identified using matlab code. Finally, the ratio of co-localized peak intensities (VGlut1/Synaptophysin) were aggregated and tau_P301L_ neg compared to tau_P301L_ pos (Figure 4).

## Data availability statement

The raw data supporting the conclusions of this article will be made available by the authors, without undue reservation.

## Ethics statement

The animal study was reviewed and approved by Auburn University Institutional Animal Care and Use Committee (IACUC).

## Author contributions

MG and MR performed the experimental design and analysis. ET managed the mouse colony breeding and sample preparation. MG, ET, MH, and JP performed the experiments. MG, ET, MH, JP, and MR analyzed the experimental results and wrote the manuscript. MG performed the computational analysis. All authors contributed to the article and approved the submitted version.
