# Supplementary material for: rTg(TauP301L)4510 mice exhibit increased VGlut1 in hippocampal presynaptic glutamatergic vesicles and increased extracellular glutamate release
Source: Front Synaptic Neurosci. 2022 Aug 3;14:925546. doi: 10.3389/fnsyn.2022.925546 (PMC9383415; doi:10.3389/fnsyn.2022.925546)
Supplement: Supplementary file 3 [file Data_Sheet_1.docx]

**Supplementary Material:**

**Animal Colony and Cell Culture Methods:**

TauP301L mice were created to represent cognitive decline, neurofibrillary tangle formation, and neuronal death that is seen in human Alzheimer’s Disease (Ramsden et al., 2005; SantaCruz K. et al., 2005). The TauP301L gene encodes for human four-repeat tau with P301L mutation (4R0N). Female TauP301L mice heterozygous for the tetracycline response element – TauP301L transgene were bred with male mice heterozygous for the activator gene: tet-off tetracycline transactivator downstream of the Ca^2+^/calmodulin kinase II promotor (CktTA) (SantaCruz K. et al., 2005). The resulting mice were known as TauP301L/CktTA. Based on PCR results, the resulting pups are designated +/+, -/-. +/-, or -/+. The first + or – denotes the presence or absence of the TauP301L pos gene. The second + or – denotes the presence or absence of the CktTA activator gene. CktTA activates TauP301L pos and creates the characteristics of Alzheimer’s Disease. Mice with both genes (+/+) were used to represent Alzheimer’s Disease and -/+, +/-, and -/- mice were used as negative controls. Since they do not have both the responder and the activator genes, the Alzheimer’s Disease phenotypes will not be displayed (Hoover et al., 2010; Ramsden et al., 2005; SantaCruz K. et al., 2005).

Around 16-20 TauP301L females and 4 CktTA males are used for consistent rotation and bred following. Females were grouped into pairs and were housed together unless they were pregnant or with a litter. The day a litter is born is designated post-natal day 0 (PND 0). On PND 3 or 4, the pups receive paw tattoos and tail snips to use for PCR. On PND 4 or 5, their hippocampi are dissected, and the cells are cultured for experiments.

Cell cultures were plated on glass coverslips in 12-well plates made. One week prior to dissections two 12-well plates were plated with a uniform confluent layer of astrocytes. One plate contained only +/+ astrocytes (Plate 1, which we call tau_P301L_ pos). A second separate plate (Plate 2) contained +/-, -/+, -/- astrocytes combined (which we call tau_P301L_ neg). All animal dissections occurred on PND 4 or 5 and followed previously established protocols, and in accordance with approved IACUC protocols. All +/-, -/+, -/- pups were dissected, combined, and plated at the same time on Plate 2 (which we call tau_P301L_ neg). All +/+ pups were dissected separately and plated on the +/+ only astrocytes in Plate 1 (which we call tau_P301L_ pos). All neurons, regardless of condition, were plated at a density of 20k per plate. 12 hours after plating cell media was changed to a neurobasal medium for neuronal growth.

Plated cells were transfected following established pHluorine-VGlut1 lentiviral vector methods(Maschi et al., 2018, 2021; Maschi & Klyachko, 2017; Voglmaier et al., 2006). Briefly, cells were transfected with 1mL of virus+Neurobasal medium with MOI ~1 at 3DIV. Cell media was then changed to back to neurobasal growth medium after 48 hrs of virus exposure. Every 5 days half the media was replaced with fresh neurobasal media. Cells were maintained in a cell culture incubator before imaging between 13 – 18 days.

For iGluSnFR experiments, plated cells were transfected with the pAAV.hSynapsin.SF-iGluSnFR.A184S (Addgene) vector, following established AAV viral vector methods (Marvin et al., 2018). Briefly, cells were transfected with 1mL of virus+Neurobasal medium with MOI ~40 at 3-4DIV. Cell media was then changed to back to neurobasal growth medium after 48 hrs of virus exposure. Every 5 days half the media was replaced with fresh neurobasal media.Cells were maintained in a cell culture incubator before imaging between 13 -18 days.

**Experimental Approach and Analysis Methods:**

**Immunocytochemistry**

Immunocytochemistry was performed similar to previously described (Glynn et al 2006). Briefly, media was removed from coverslips of healthy neurons were fixed with a solution of cold 4% paraformaldehyde in 0.1M phosphate buffered saline (PBS) with 4% sucrose for 10 minutes at 4°C. The fixative was quenched by washing with 0.1M glycine for 5 minutes and then washed three times. Cell membranes were permeabilized with 0.25% Triton X-100 for X min and samples were washed. Nonspecific binding was then blocked with incubation in 10% Normal Goat serum for 30 minutes. Primary antibodies were added and incubated in 1% Normal Goat serum in 0.1M PBS at 4°C overnight. Cells were then washed before secondary antibody incubation for 2 hours. Cells were washed and a DAPI counterstain was added for 5 minutes. Cells were washed one more time and mounted onto gelatin coated slides with Permount and fixed. All steps were performed at room temperature on a shaker at 40 rpm in the dark unless otherwise noted. All solutions were made using 0.1M PBS diluent. Samples were imaged on a Nikon Ti2 Fluorescence microscope.

**Experimental Setup for pHluorin and iGluSnFR**

Samples were imaged on a custom built microscope based on a Nikon Ti2-E inverted microscope base (Nikon), surrounded by a temperature controlled incubation chamber held at 37C (OKOLab). Samples were illuminated using an LED source (SOLA), with excitation and emission frequencies filtered using a GFP cube (Nikon). Intensity was focused onto samples using a 100x oil objective (Nikon). Emitted light intensity was collected using an Orca Flash v4 CMOS camera (Hamamatsu). Images were taken at 80 msec exposure followed by a 20 msec readout time. Images were recorded using Hamamatsu software in *.cxd format and converted to individual *.tif files later for analysis (Hamamatsu). Field stimulation was applied using a square-pulse stimulator (BKPrecision 4030 10 MHz generator), with 1msec pulse durations at 10V/cm. Samples were kept alive during experiments and perfused using a multi-channel perfusion system (World Precision Instruments) with a modified tyrodes solution pH-balanced to 7.4 following previously established protocols. Timing and control of all equipment was performed using a Marter9 TTL controller (AMPI Instruments).

Specifically, we chose an initial stimulation frequency of 40Hz in order to drive a significant portion of the recycling pool to release in order to observe a difference in VGlut1-pHluorin intensity in tau_P301L_ pos neurons as compared to tau_P301L_ neg controls. Natural spike trains in hippocampal neurons range from 1 Hz up to ~100 Hz (Klyachko & Stevens, 2006). However, hippocampal neurons exhibit an increase in spiking rates characterized by extended bouts (~10 sec) of higher-frequency spiking rates ( >=20 Hz) prior to neurodegeneration in AD models (Kazim et al., 2017). Consequently, a single 10 sec bout of electrical stimulation at a constant rate of 40 Hz will model this hyperexcitable state and allow for a clear observable difference in VGlut1-pHluorin intensity (Fig. 1B).

**Bulk pHluorin Analysis:**

Bulk pHluorin intensity curves were obtained from aggregating individual presynapses using the same process previously used for dye loading/unloading (Gramlich & Klyachko, 2017; Maschi et al., 2021). Briefly, raw tiff files were background subtracted using a 30-pixel rolling ball radius in ImageJ; second, single presynapses were identified and integrated using a 15x15 pixel box in custom written Matlab code; third, each presynapse pHluorin curve was background subtracted so that all curves were zero intensity just before stimulation (10 sec, see Fig. 1); then, the counts for each presynapse was binned for all curves as a function of frame and fit to a gaussian curve; Finally, the resulting mean +/- SEM as a function of frame was plotted for each condition.


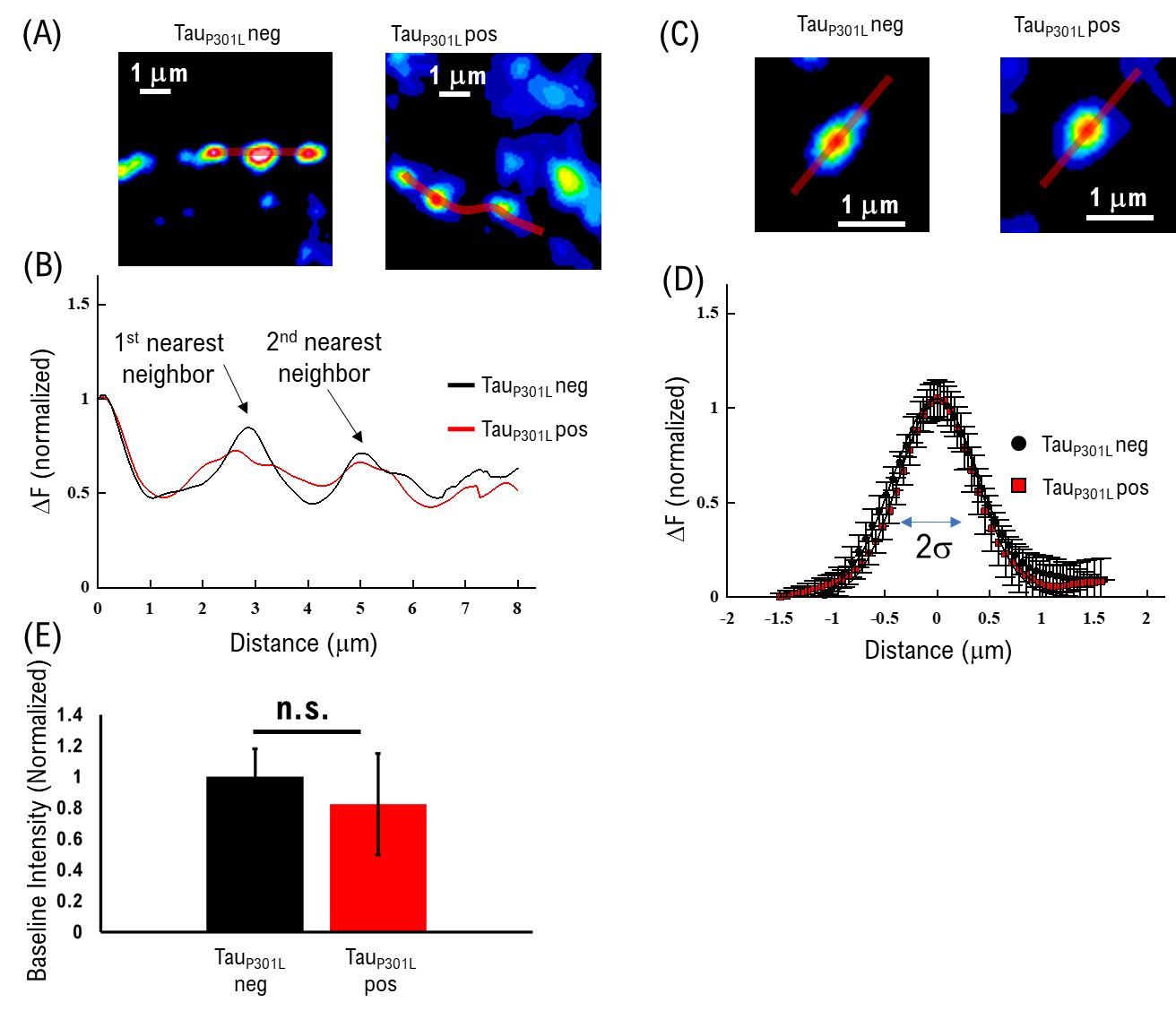


**Figure S1: Synapse Density, Size, and Baseline Intensity:**

(A) Example of intensity integration line used to determine distance between presynapses

(B) Aggregated integrated VGlut11-pHluorin intensity (Red line indicated in (A)) for tau_P301L_ pos (Red, N = 12) and tau_P301L_ neg (Black, N = 10). Nearest neighbor peaks are indicated.

(C) Example of intensity integration line used to determine width of presynapses

(D) Aggregated integrated pHluorin-VGLUT1 line intensity of individual presynapses for tau_P301L_ pos (Red, N = 12) and tau_P301L_ neg (Black, N = 10).

(E) Mean aggregate baseline intensity for all conditions prior to electrical stimulation for tau_P301L_ pos (Red, N = 920, from 9 samples from 8 litters) and tau_P301L_ neg (Black, N = 907 rom 8 samples from 6 litters).

n.s. = not significant from two-tailed t-Test of distribution.

To determine if the density of presynapses changes in tau_P301L_ pos compared to tau_P301L_ neg, we used integrated intensity line analysis. We used pHluosin-VGlut1 intensity data after samples were exposed to NH_4_Cl, causing a complete unloading of all presynapses (Ganguly et al., 2015). We used ImageJ to open single raw tiff files at the frame of peak intensity (Fig. S1 A). We drew a line along an axon starting at a presynapse and ending just after a third neighboring presynapse. We then normalized the VGlut1-pHluorin intensity to the first presynapse. Finally, we aggregated multiple lines to determine an average intensity as a function of distance from the first presynapse (Fig. S1 B). We found that the VGlut1-pHluorin intensity decreased as a function of distance and begin to increase again until reaching a peak the first nearest neighbor (1^st^ nearest neighbor, Fig. S1 B). The VGlut1-pHluorin intensity then began to decrease again, and then rise again peaking at the second nearest neighbor (2^nd^ nearest neighbor, Fig. S1 B). The nearest neighbor peaks occurred at approximately the same location for tau_P301L_ pos (2.6 μm) compared to tau_P301L_ neg (2.86 μm). We note that there was a slight shift in the tau_P301L_ pos distance between presynapses, as well as an increase in the distribution of distances, but the difference did not significantly alter the density of presynapses along the axon. Further, these distributions are consistent with our previously observed distributions for rat hippocampal cell cultures (Gramlich & Klyachko, 2017).

Separately, we sought to determine if the overall size of the presynapse changed in tau_P301L_ pos compared to tau_P301L_ neg, which would potentially alter the total amount of VGlut1-pHluorin intensity. To determine this possibility we used the same VGlut1-pHluorin line intensity integration above, but for single presynapses. We used ImageJ to open single raw tiff files at the frame of peak intensity (Fig. S1 C). We drew a line along an axon across a presynapse. We then normalized the VGlut1-pHluorin intensity to the first presynapse. Finally, we aggregated multiple lines to determine an average VGlut1-pHluorin intensity across a presynapse (Fig. S1 D). We found that the VGlut1-pHluorin intensity FWHM (2σ, Fig. S1 D) was slightly smaller for tau_P301L_ pos (0.76 μm) compared to tau_P301L_ neg (0.82 μm). However, these differences are not significant compared to the uncertainty in the data, nor would the difference significantly contribute to the observed differences in overall VGlut1-pHluorin intensity observed.

One possible reason tau_P301L_ pos neurons result in a 40% increase in intensity is that tau_P301L_ pos vesicles have a 40% lower pH-value. When exposed to the neutral pH of the extracellular environment, tau_P301L_ pos vesicles would increase their intensity 40% than tau_P301L_ neg vesicles because they started from a lower value. Our baseline normalization would then mask this difference and result in a 40% increase observed (Fig. 1). To control for this possibility, we aggregated raw baseline intensities for all high-frequency Vglut1-pHluorin data (10 Hz, 20 Hz, 40 Hz) for 1 second prior to stimulation. We then obtained a gaussian mean and variance from fits to histograms of the data for tau_P301L_ neg and tau_P301L_ pos data (Fig. S1E). We observed a slightly lower (<20%) overall baseline intensity in tau_P301L_ pos neurons compared to tau_P301L_ neg, but the reduction was not statistically significant (P = 0.62). Further, the lower overall baseline was less than the observed increase (40%), which would support our hypothesis that the number of VGlut1 transporters per vesicle causes the difference in observed intensities.

**Computational Simulation Approach for Bulk VGlut1-pHluorin Experiments:**


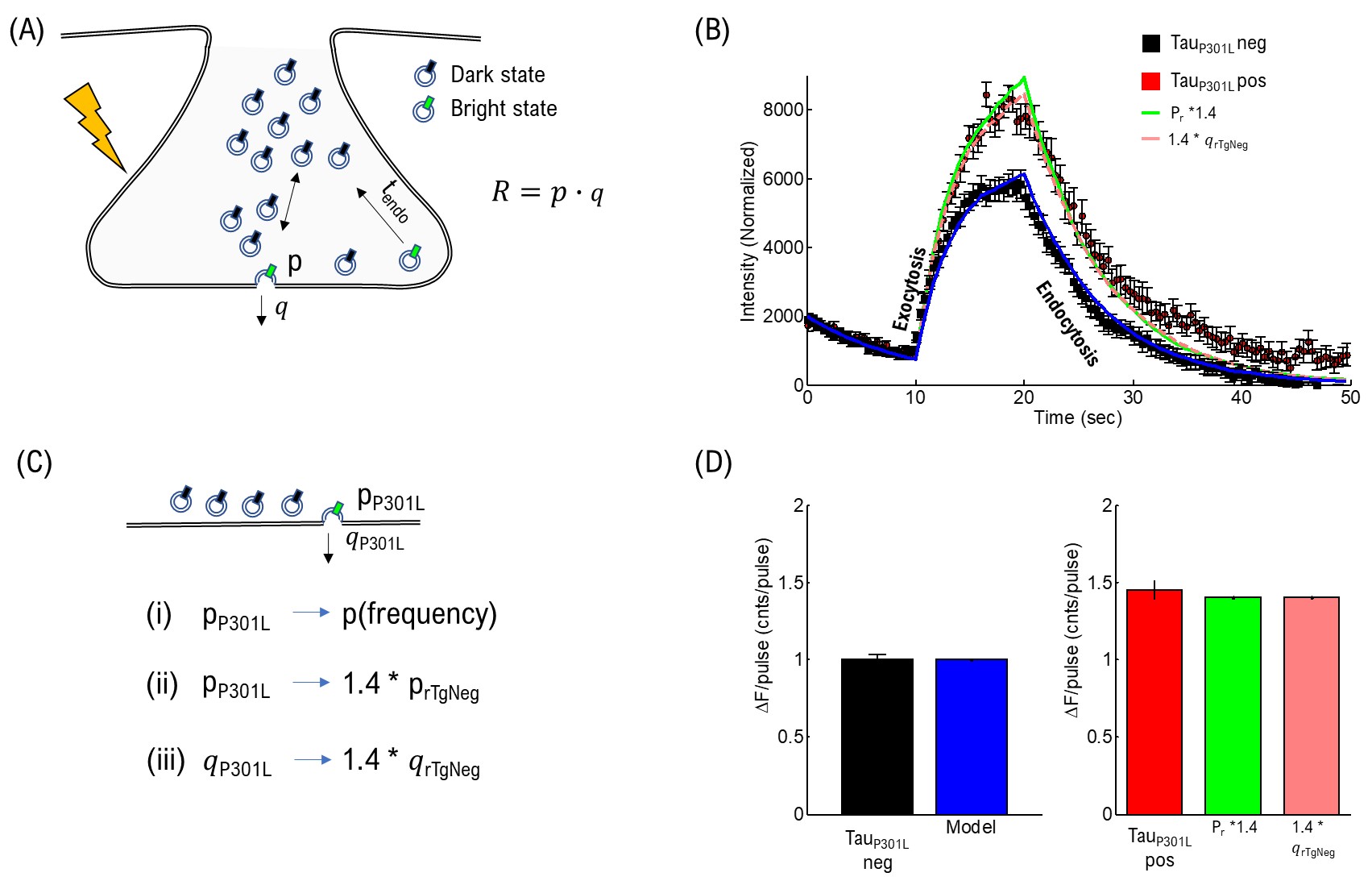


**Figure S2: Computational model of presynaptic exocytosis**

(A) Cartoon representation of computational model of presynaptic release in tau_P301L_ neg and tau_P301L_ pos neurons

(B) Comparison of pHluorin data and computational model

(C) Hypothesized pathways for tau_P301L_ pos pHluorin-VGLUT1 intensity increase

(D) Quantitative comparison of resulting ΔF/pulse for experimental results and computational models

Computational simulations were performed using a model based on the established binomial vesicle release model, as shown in eqns. {2}, {3}. All model parameters used to reproduce each experimental conditions were constrained (Table S1) using the tau_P301L_ neg 40 Hz data (Black circles Fig. S2 B). Only the intensity per vesicle (q) and release probability (p_0_) were allowed to change in order to match observed rTgPos 40 Hz condition (Red Squares Fig. S2 B). Further, we modeled an exponentially decaying vesicle release probability with time during stimulation ( $p\left( t \right)=p_{0}+\Delta p*e^{-t/\tau_{p}}$ ) which course-grains a combination of factors that have been extensively explored previously, but are beyond the scope of this present study. The same time-dependent reduction response was used for both tau_P301L_ neg and rTgPos conditions and reproduced the same intensity curves.

| Parameter | Value | Purpose |
| --- | --- | --- |
| Pulse/frame | 4 | Number of stimulation pulses per each frame |
| τ_p_ | 7 (sec) | Rate of reduction in release probability during stimulation |
| Δp | 1.6 | Release probability pre-factor |
| Stim start | 10 (sec) | Time stimulation starts |
| Stim end | 20 (sec) | Time stimulation ends |
| τ_endo_ | 7 (sec) | Endocytosis Rate |
| BKG_start | 1000 (cnts) | Background Intensity at start of simulation |
| BKG_end | -2000 (cnts) | Background Intensity at end of simulation |
| τ_BKG_ | 10 (sec) | Rate of decay in background intensity from photobleaching |
| PR | 0.1 | Fraction of intensity lost due to photobleaching at each time-step |
| p_0_ | free | Release probability |
| q | free | Intensity per vesicle |

Table S1: Computational Simulation Parameters

Simulations were performed following the same algorithm approach using python 3.10 release. All simulation parameters (Table S1) are fixed at the beginning of each simulation. A single array (**I**) is created with length equal to the total number of simulation time-steps. Each time-step (Δt) in our simulations were equivalent to a single frame exposure time (100 msec). Each array element (**I**(Δt)) stores the total observable intensity (background + vesicles) equivalent to counts observed experimentally. We used a dynamic Monte-Carlo approach, that we have previously used to model vesicle probabilities(Gramlich et al., 2021; Gramlich & Klyachko, 2017), to model each probabilistic process in simulations of vesicle release mechanics.

The algorithm steps for each simulation time-step were as follows:

[1] The simulation sets the initial background intensity at Δt = 0.

[2] When the simulation time-step equals the stimulation start-time (Δt = Stim start), an unweighted random number (R_1_) are chosen between 0 and 1.

[3] If R_1_ is below the current release probability ( p(Δt) ), then a single vesicle is released with intensity (q) which is added to the total intensity for the array (**I**(Δt) = **I**(Δt)+q).

[4] A random number (R_2_) is then chosen from an exponential random number generator (np.random.exponential) seeded with the endocytosis rate (τ_endo_). This number represents the number of simulation time-steps that the released vesicle in [3] is counted in the array. Array elements from the current time-step (**I**(Δt) += q) up to the randomly generated time-step (**I**(Δt + R_2_) += q) have the vesicle intensity added.

[5] The algorithm steps [2]-[4] are repeated at the same time-step (Δt) for up to the number of experimental pulses used (Pulse/frame).

[6] The total intensity for the current simulation time-step is reduced by the amount fixed by the photo-bleaching parameter (PR)

[7] Algorithm steps [2]-[6] are repeated until the simulation time-step equals the end of the stimulation (Δt = Stim end)

[8] After stimulation, the remaining simulation time-steps reduce the intensity of each element by the amount fixed by the photo-bleaching parameter (PR)

To reproduce averaged VGlut1-pHluorin intensity results, we simulated 100 presynapses and reported the averaged simulated intensity. For each hypothetically proposed pathway, (ii)-(iii) in section 2.2, we ran 100 simulations. We then averaged the resulting intensity for all 100 simulations, and compared the averaged intensity with experimental results (Fig. S2 B).


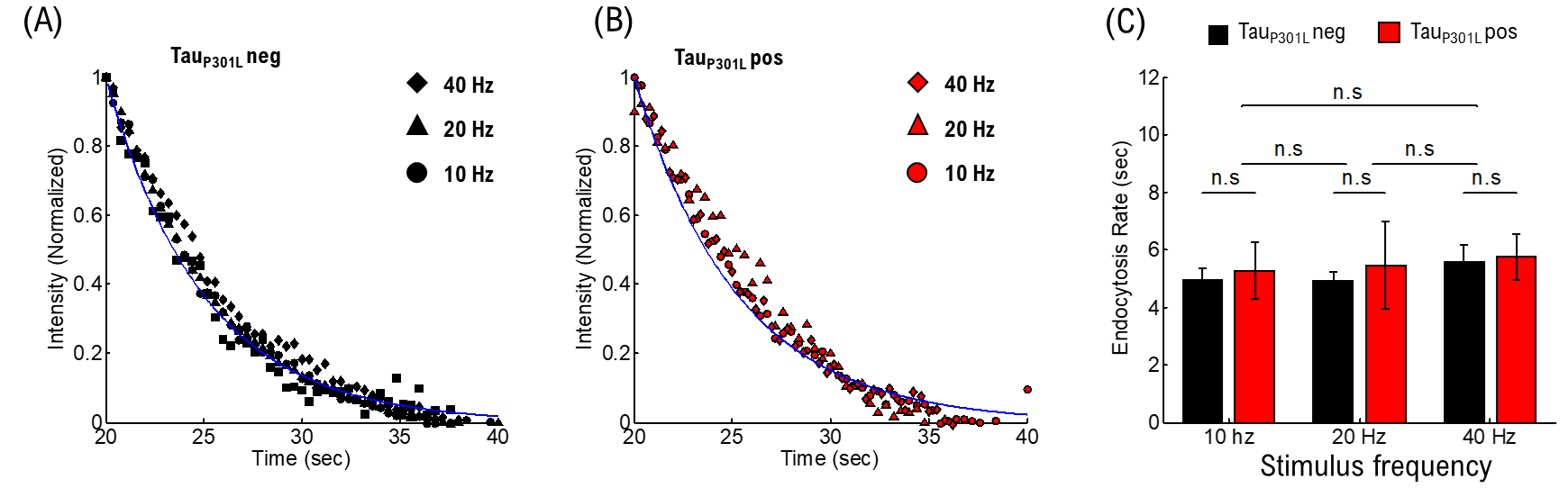


**Figure S3: Endocytosis Rate does not contribute to increases in tau_P301L_ pos pHluorin-VGLUT1 intensity**

(A) Comparison of pHluorin-VGLUT1 intensity decay after stimulation (20-40 sec) as a function of stimulation frequency for tau_P301L_ neg neurons.

(B) Comparison of pHluorin-VGLUT1intensity decay after stimulation (20-40 sec) as a function of stimulation frequency for tau_P301L_ pos neurons.

(C) Endocytosis Rate from exponential decay fits (Blue Line in D,E). Each curve fit separately.

10 Hz: tau_P301L_ neg N = 134 presynapses, 1 sample from 1 litters; tau_P301L_ pos N = 193 presynapses, 2 samples from 2 litters

20 Hz: tau_P301L_ neg N = 615 presynapses, 5 samples from 3 litters; tau_P301L_ pos N = 151 presynapses, 3 samples from 2 litters

40 Hz: tau_P301L_ neg N = 257 presynapses, 3 samples from 2 litters; tau_P301L_ pos N = 160 presynapses, 3 samples from 2 litters

Errors are twice the 95% confidence interval value of fits to data. *** = P<0.01; Statistical results from Mann-Whitney U.

To quantify the difference between tau_P301L_ neg and tau_P301L_ pos intensity as a function of stimulation frequency, we fit the frequency-dependent curves to our continuum model (eqn. 1, Blue Lines in Fig. S2 A,B). Each curve was fit with the same constraints on endocytosis rate and background intensity, resulting in a single fit parameter corresponding to the difference in single vesicle intensity per pulse (ΔF/pulse). Lastly, to directly compare changes as a function of frequency, we normalized the ΔF/pulse to the tau_P301L_ neg parameter (Fig. S3 C).

**Single Vesicle VGlut1-pHluorin Analysis:**


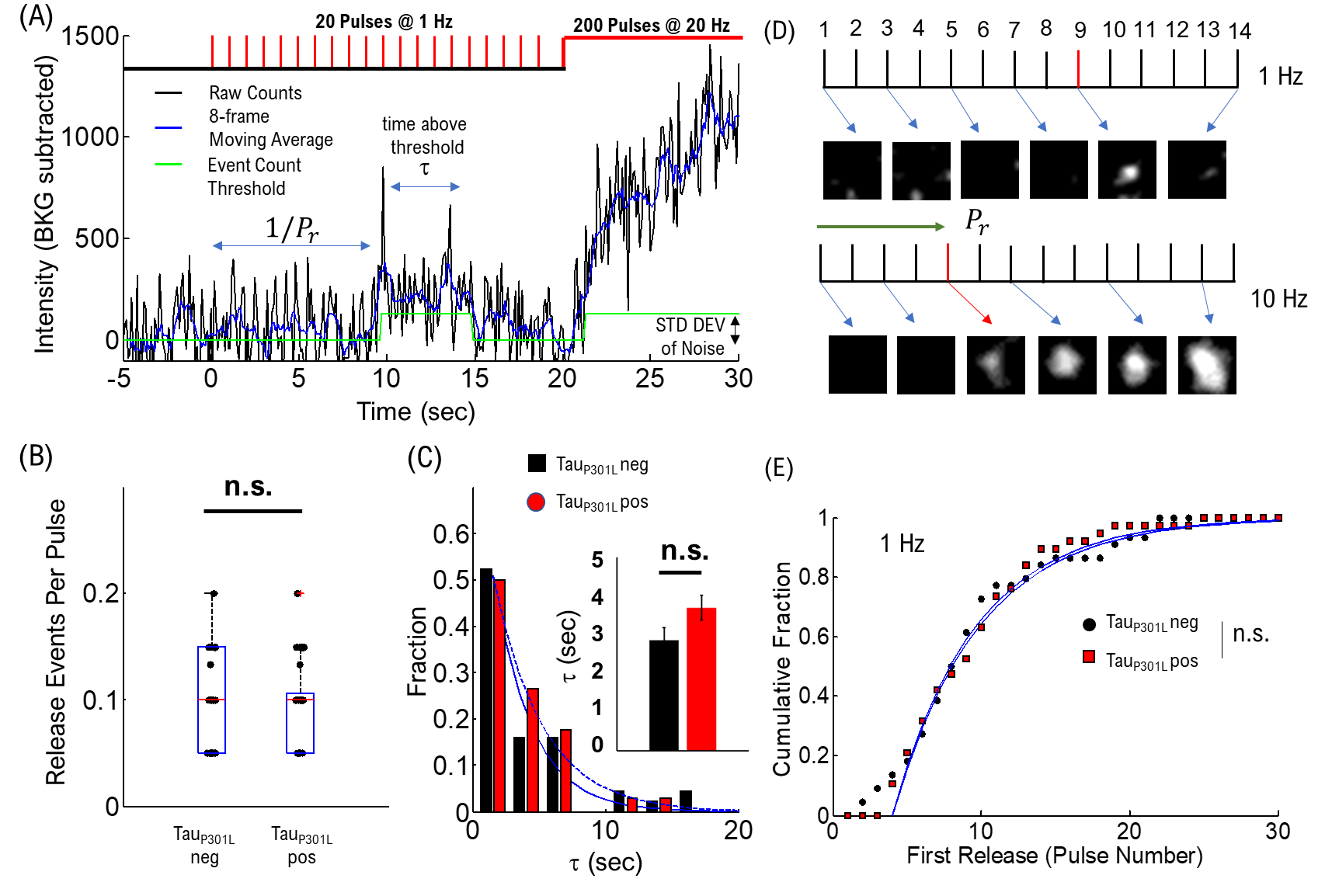


**Figure S4: Single Vesicle Release Event Analysis Method**

(A) Example intensity trace and analysis approach as a function of time for tau_P301L_ neg. Integrated intensity (Box in Fig. 3A) is background subtracted (Black), along with the moving average (Blue), and the single release event count threshold (Green).

(B) Aggregate number of release events divided by number of pulses for tau_P301L_ neg and tau_P301L_ pos conditions. Mean ± Standard-Deviation.

(C) Aggregate histogram distributions for time spent above threshold (τ) for tau_P301L_ neg and tau_P301L_ pos conditions, along with exponential fits to data (Blue lines). Inset shows exponential fit-values for mean time above threshold.

Statistical tests are two-tailed t-Test of distributions.

1 Hz: tau_P301L_ neg N = 33 vesicles, 2 samples from 2 litters; tau_P301L_ pos N = 37 vesicles, 3 samples from 2 litters

(D) Example Initial Vesicle Release events as a function of Pulse Number for tau_P301L_ neg and tau_P301L_ pos conditions for 1 Hz and 10 Hz.

(E) Aggregate cumulative distribution analysis of number of pulses until the first release event is observed for 1Hz for tau_P301L_ neg (black) and tau_P301L_ pos conditions (red).

All values reported as mean ± SEM. Statistical tests are two-tailed KS-test of cumulative fractions.

Single vesicle events were determined following previously established Vglut1-pHluorin intensity analysis approaches (Chanaday & Kavalali, 2018). First, single vesicle data were experimentally obtained by an initial bout of low-frequency stimulation (1 Hz or 2 Hz) followed by a second bout of higher stimulation (20 Hz or 40 Hz). Presynapses that exhibited intensity during high frequency stimulation were selected for analysis for single vesicle events. Second, selected presynapses were background subtracted using a 10-pixel rolling ball radius in ImageJ, followed by a 1-pixel Gaussian blur in ImageJ. Third, single vesicle intensity was determined from a 15x15 pixel integration box (Dashed Box Fig. 3A) from each identified presynapse location (Black line, Fig. S4 A). Integrated intensity was then run through an 8-frame moving-average filter (Blue Line Fig. S4 A), which we have previously established as a roboust approach (Gramlich & Klyachko, 2017; Maschi et al., 2018, 2021), but resulted in the same release probability results obtained using more complex filtering algorithms by other groups (Chanaday & Kavalali, 2018). Fourth, a simple threshold analysis was used where release events were counted when the integrated intensity was greater than a threshold value equivalent to the standard deviation of the noise (130 cnts/frame, Double Arrows Fig. S4 A) for at least 500 msec (Green line Fig. S4 A). Finally, the total time the integrated intensity was greater than the standard deviation of the noise threshold was counted (τ, Fig. S4 A).

We also used the single vesicle threshold analysis to determine the number of release events per pulse as an estimate of the release probability (Fig. 2 F, Fig. S4 B). First, an event was counted each time the intensity was above the threshold for 1 Hz stimulation frequency (Green Line, Fig. S4 A). Whenever the intensity returned to background for at least 500 msec and then rose above threshold intensity a new event was counted. Second, the number of unique release events were then divided by the number of pulses. Third, the distribution of the number of release events per pulse compared (Fig. S4 B), which were used to determine the average value and standard deviation as an estimate of the release probability (Fig. 2F). Both the time to first release and the number of release events per pulse result in the same estimate of the release probability (P_r_ = 0.1).

We last estimated the amount of time each unique release event was observed above the threshold value at 1 Hz stimulation. To determine the time above threshold (τ, Fig. S4 A), we only counted events where the intensity returned to background before the onset of high-frequency stimulation, which excluded approximately 30% of all release events observed. We then compared the exponential fits to histograms (Blue lines Fig. S4 C) of event times for tau_P301L_ neg (Black Fig. S4 C) tau_P301L_ pos (Red Fig. S4 C). The mean time was slightly longer for tau_P301L_ pos (3.7 +/- 0.3 sec, inset Fig. S4 C) than tau_P301L_ neg (2.9 +/- 0.3 sec, inset Fig. S4 C), but was not statistically significant (P = 0.2, two-tailed t-Test). Further, these times are consistent with previously observed dwell-times for single release events using VGlut1-pHluorin analysis (Chanaday & Kavalali, 2018; Leitz & Kavalali, 2011).

We used the single vesicle threshold analysis to determine the number of pulses until release (Fig. 2 D,E) as an estimate of the release probability. First, the number of pulses were counted until the intensity was above the threshold when a release event was identified for low stimulation frequencies 1 Hz, 2Hz, and 10 Hz (Fig. 2 D , Fig. S4 D). The cumulative distribution of the number of pulses were then fit to determine the mean number of pulses until release (Fig. S4 E). Finally, the inverse of the number of pulses and SEM were used to determine the release probability for all frequencies (Fig. S4 E).

**Single Vesicle Vglut1-pHluorin Point-Spread-Function and Integrated Intensity Variance:**


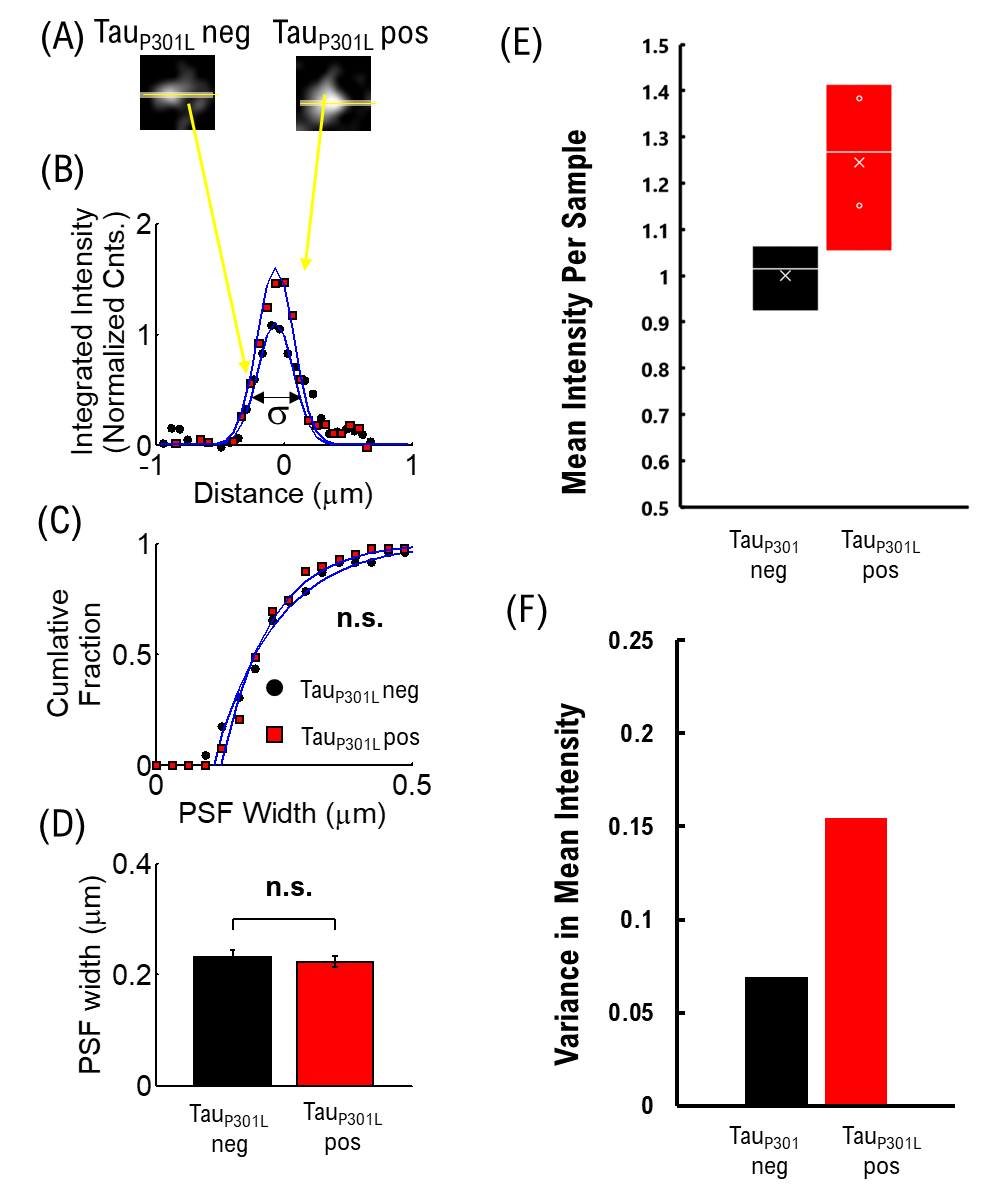


**Figure S5: Variance in Intensity Across Samples**

**Figure S5: Quantized single vesicle VGlut1-pHlluorin-VGLUT1 point-spread-function and integrated intensity Variance**

(A) Example single vesicle events and line-profile analysis (Solid Yellow lines) for tau_P301L_ neg and tau_P301L_ pos neurons.

(B) Example PSF intensity of tau_P301L_ neg (Black circles) and tau_P301L_ pos (Red squares) neurons from line-profiles in (A). Intensity has been background subtracted and normalized to tau_P301L_ neg peak intensity. Single gaussian fits to data (Blue solid lines), with PSF-width (σ) used to determine MVR.

(C) Cumulative fraction distributions of tau_P301L_ neg (Black circles, N = 22) and tau_P301L_ pos (Red squares, N = 38) of single vesicle PSF-width (σ in B). Cumulative distributions fit to a single cumulative fit function (Blue Lines).

(D) Mean values from cumulative fits to data (Blue lines in C), with errors from 95% confidence intervals of fits.

(E) Average single vesicle integrated intensity (Fig. 3A) for different samples measured normalized to average tauP301L neg intensity for all samples combined.

(F) Variance in mean single vesicle integrated intensity across samples.

tauP301L neg N = 5 samples, from 4 litters; P301L N = 4 samples from 4 litters

To establish that single vesicle releases are the predominant process in tau_P301L_ pos, we quantified the point-spread-function (PSF) per release event by obtaining line-profile counts for vesicles during the first observed frame (Fig. S5 AB). We fit the PSF line profile to a gaussian function (Blue lines in Fig. S5 B) to determine the width of each observed release event (σ, Fig. S5 B). If multiple vesicles were released at the same time in tau_P301L_ pos as compared to tau_P301L_ neg, then the PSF-width increase (Maschi et al., 2021). We then aggregated the PSF of release events where only background intensity was observed before the release and where a single intensity step increase was observed within a 5-frame window (Fig. S3 A) in order to prevent bias of asynchronous or spontaneous release events. These restrictions resulted in a limited but comparable number of events for both tau_P301L_ pos (N = 38) and tau_P301L_ neg conditions (N = 22).

To estimate the variance in single vesicle intensity across samples and litters, we quantified the average intensity for each sample and compared the distribution of averages normalized to the tau_P301L_ neg condition (Fig. S5 E), as well as the variance in average intensities across samples (Fig. S5 F). The distribution of tau_P301L_ neg intensities was lower than for tau_P301L_ pos samples consistent with the result from aggregate analysis (Fig. 3 B,C). Further, the variance in intensities across samples doubled for tau_P301L_ pos compared to for tau_P301L_ neg (Fig. S5 F), which is consistent with the increase in variance observed from aggregate analysis (Fig. 6 C,E). However, one limitation to the average analysis across samples is that some samples contain fewer numbers of identified vesicles increasing the uncertainty per sample, and lowered the overall average tau_P301L_ pos intensity.

**Bulk iGluSnFR Analysis:**

Bulk iGluSnFR intensity curves were obtained from aggregating individual presynapses using the same process previously used for pHluorin intensity. Briefly, raw tiff files were background subtracted using a 30-pixel rolling ball radius in ImageJ; second, single presynapses were identified and integrated using a 15x15 pixel box in custom written Matlab code; third, each presynapse pHluorin curve was background subtracted so that all curves were zero intensity just before NH4Cl exposure (50 sec, see Fig. 5); then, the mean peak intensity was determined by averaging the 10 frames with the most intensity for each presynapse; Finally, the resulting mean +/- SEM as a function of frame was plotted for each condition.

**Variance in Synaptophysin expression measured in tau_P301L_ pos and tau_P301L_ neg:**

To determine if the synaptophysin distribution changed in immunohistochemistry analysis of tau_P301L_ pos compared to tau_P301L_ neg, we compared the distribution of intensities between samples within a litter and across litters. Immunohistochemistry data included 2-3 plates for each litter. We aggregated the distribution for all plates in a litter and used the width of the distribution (σ) as the variance between samples in a litter (Fig. S6 A,C). We then compared the SEM across three litters used for each condition to determine changes between litters (Fig. S6 B,D). We found that all litters had a variance less than 4% for both tau_P301L_ pos and tau_P301L_ neg.


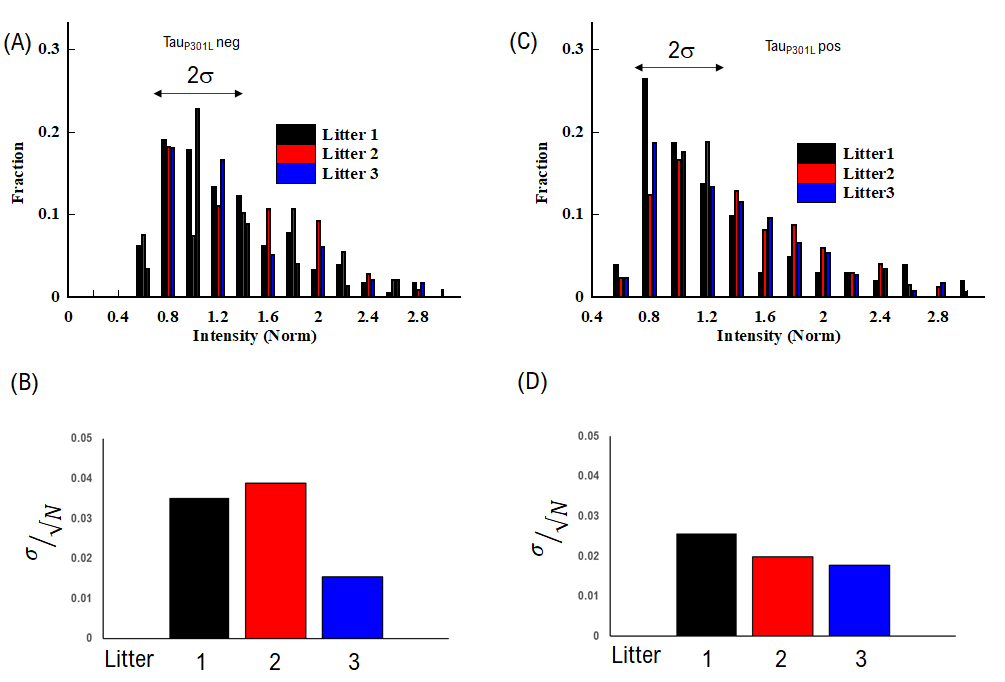


**Figure S6: Variance in Synaptophysin measured with immunohistochemistry**

(A) Distribution of Synaptophysin intensities normalized to mean across three litters measured for tau_P301L_ neg

(B) SEM measured for distribution of synaptophysin intensities for tau_P301L_ neg

(C) Distribution of Synaptophysin intensities normalized to mean across three litters measured for tau_P301L_ pos

(D) SEM measured for distribution of synaptophysin intensities for tau_P301L_ neg

**Statistical Analysis Methods:**

For single vesicle width analysis, we performed a power analysis in Matlab for a t-test statistic assuming a tau_P301L_ neg = 250 +/- 50 nm baseline distribution to determine the number of samples required to for a tau_P301L_ pos = 300 +/- 50 nm distribution. For a power of 0.99, the analysis recommended a minimum of 21 samples per distribution. Thus, both the tau_P301L_ neg and tau_P301L_ pos sample numbers were sufficient to distinguish the difference if it existed.

Best fit to the number of VGlut1 transporters per vesicle were determined using a sum of residuals squared. Briefly, the fits involved a pair of curves for each condition (i.e. one curve for 4 channels fit to tau_P301L_ neg and one curve for 5 channels fit to tau_P301L_ pos). The residuals for each pair were calculated as:

$$R^{2}=\sum{(\mathrm{tau}P301L\mathrm{neg}\left( x \right) - y_{1}(x))}^{2}+{(\mathrm{tau}P301L\mathrm{pos}\left( x \right) - y_{2}(x))}^{2}$$

where y_1_ is the fit curve for tau_P301L_ neg and y_2_ is the fit curve for tau_P301L_ pos.

The lowest total R^2^ value determined the best fit to the data.
